# Controlled formation of versatile methylated compounds based on ring opening of 4-methyl-1-siloxy-1,4-epoxy-1,4-dihydrobenzene

**DOI:** 10.1039/d6ra01853j

**Published:** 2026-03-19

**Authors:** Takaaki Aijima, Jin Tokunaga, Sota Yoshimura, Yuki Itabashi, Tsunayoshi Takehara, Takeyuki Suzuki, Shuji Akai, Yoshinari Sawama

**Affiliations:** a Graduate School of Pharmaceutical Sciences, The University of Osaka 1-6 Yamada-oka Suita Osaka 565-0871 Japan; b Institute for Open and Transdisciplinary Research Initiative (OTRI), The University of Osaka 1-6 Yamada-oka Suita Osaka 565-0871 Japan; c SANKEN, The University of Osaka 8-1 Mihogaoka Ibaraki Osaka 567-0047 Japan

## Abstract

We report an FeCl_3_-catalyzed transformation of 4-methyl-1-siloxy-1,4-epoxy-1,4-dihydrobenzene. Reaction in toluene gave the phenol product, whereas the addition of *i*-PrOH in 1,2-dichloroethane induced desilylative ring opening to produce 4-hydroxy-4-methyl-2,5-cyclohexadienone, which subsequently underwent a CO_2_Me-induced regioselective 1,2-methyl shift (C4 to C3) to afford 6-methyl-2,4-cyclohexadienone. This product bears a methyl-substituted quaternary carbon center that is difficult to access by existing methods and serves as a versatile intermediate for further structural elaboration. These results highlight a new mode of skeletal rearrangement and demonstrate regioselective control over competing reaction pathways.

## Abstract 2

We report an FeCl_3_-catalyzed transformation of 4-methyl-1-siloxy-1,4-epoxy-1,4-dihydrobenzene.

## Graphical abstract

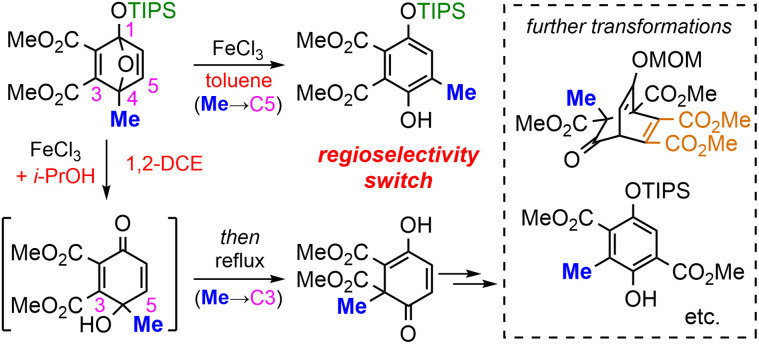

## Introduction

Skeletal rearrangements are powerful transformations in organic synthesis because they enable rapid changes in molecular connectivity from readily accessible precursors, often providing efficient access to structurally complex and synthetically challenging frameworks. From this perspective, developing new rearrangement modes and achieving regioselective control over competing pathways remain important challenges in synthetic chemistry.^1^

**Scheme 1:**
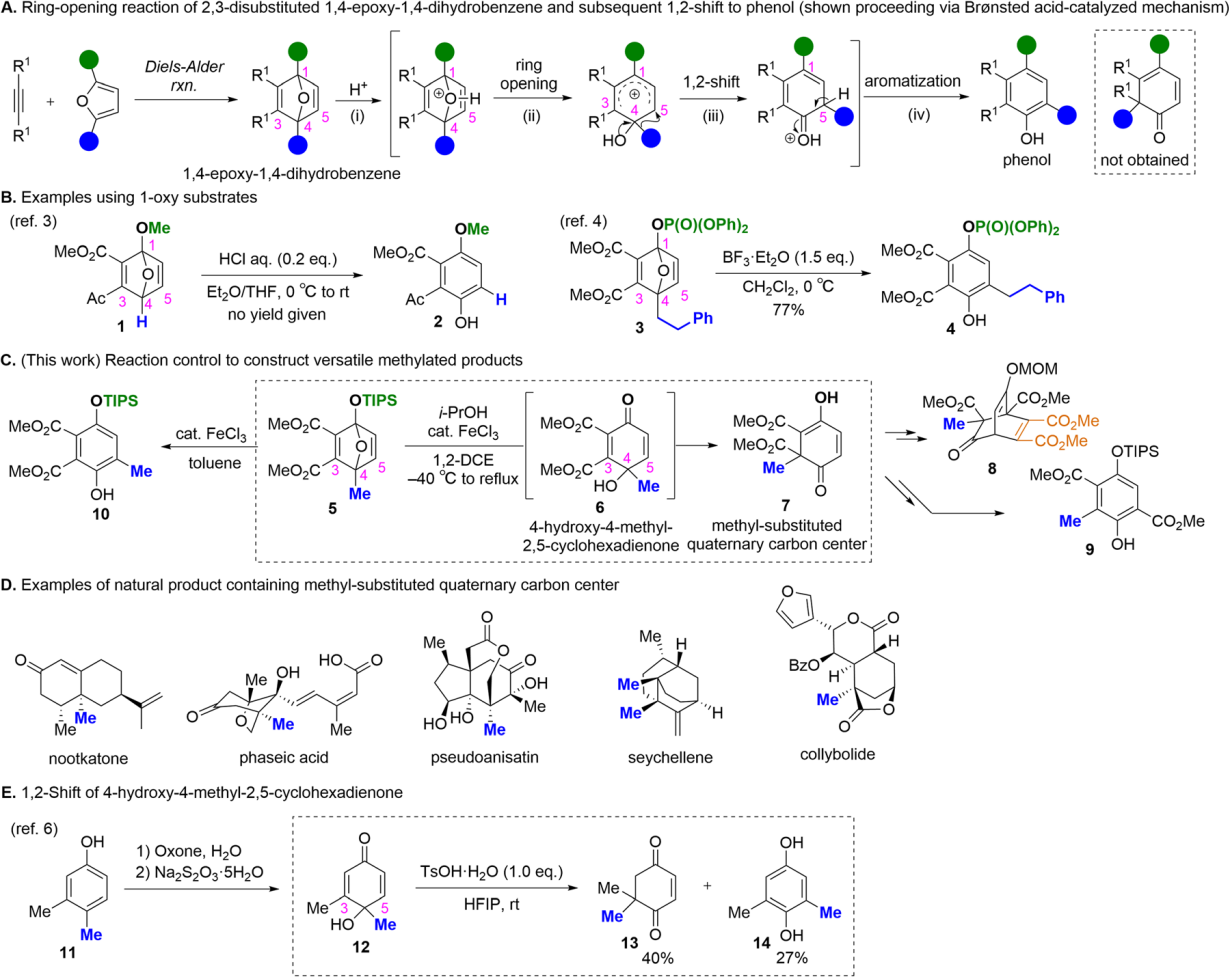
Ring opening of 1,4-epoxy-1,4-dihydrobenzene derivatives and examples of natural product containing methyl-substituted quaternary carbon center.

1,4-Epoxy-1,4-dihydrobenzene derivatives, which are readily synthesized *via* a Diels–Alder reaction between the corresponding disubstituted alkyne and furan, represent valuable scaffolds for skeletal rearrangements (Scheme 1A). Under acidic conditions, these substrates generally undergo the ring opening of the 1,4-epoxy moiety to afford a phenol derivative. This transformation proceeds through the following steps: (i) activation of the oxygen atom on the 1,4-epoxy moiety by protonation (when using a Brønsted acid), (ii) ring opening of the 1,4-epoxy moiety, (iii) a 1,2-shift of the substituent at the C4 position of substrate to the C5 position, and (iv) aromatization. These transformations are driven by the aromatic stabilization of the phenol product; therefore, products derived from a 1,2-shift to the C3 position are not generated.^2^ When using unsymmetrical substrates bearing the 1-oxy group, regioselective ring opening preferentially occurs at C1 owing to the instability of the strained cyclic acetal moiety, followed by an analogous 1,2-shift and aromatization to yield phenol products (Scheme 1B). For example, 1-methoxy substrate 1 and 1-phosphoryloxy substrate 3 were transformed into the phenol products 2 and 4, respectively, without the cleavage of the O–Me or O–P bonds in the presence of catalytic amounts of Brønsted acids or stoichiometric Lewis acids.^3,4^ Notably, a 1,2-shift of the C4 substituent toward C3, accompanied by ring opening, has not been previously achieved. Consequently, rearrangement pathways that do not generate phenol products remain largely unexplored.

Herein, we demonstrate a regioselective 1,2-shift of the methyl group at the C4 position of 4-methyl-1-siloxy-1,4-epoxy-1,4-dihydrobenzene 5 to C3, *via* FeCl_3_-catalyzed desilylative ring opening (Scheme 1C). In 1,2-dichloroethane (1,2-DCE), treatment of 5 with catalytic FeCl_3_ in the presence of *i*-PrOH as an additive induced desilylative ring opening to yield 4-hydroxy-4-methyl-2,5-cyclohexadienone 6, which then underwent a regioselective 1,2-shift of the methyl group at C4 to C3, affording 6-methyl-2,4-cyclohexadienone 7. The present methodology enables the construction of a methyl-substituted quaternary carbon center, a motif frequently found in natural products that remains challenging to access synthetically (Scheme 1D).^5^ Recently, Lawrence and co-workers reported a 1,2-shift of the methyl group at the C4 position of 4-hydroxy-4-methyl-2,5-cyclohexadienone 12, prepared from phenol derivative 11, to the C3 position in the presence of stoichiometric TsOH·H_2_O in hexafluoroisopropanol (HFIP), affording 13 along with the typical phenol product 14 without regioselective control (Scheme 1E).^6^ In contrast, the present work enabled regioselective control of the 1,2-shift of the methyl group, and can be extended to access other methylated products such as 8 and 9. Notably, the treatment of 5 with FeCl_3_ gave the typical phenol product 10.

## Result and discussion

First, 4-methyl-1-triisopropylsiloxy-1,4-epoxy-1,4-dihydrobenzene 5 was prepared from dimethyl acetylenedicarboxylate and 4-methyl-1-(triisopropylsiloxy)furan (see the SI for details). The ring opening of 5 was investigated using FeCl_3_ as an inexpensive and versatile Lewis acid^7^ (Table 1A). We hypothesized that the 1,4-epoxy moiety of 5 would be activated by FeCl_3_ and that the strained acetal moiety would be preferentially cleaved to yield reaction intermediate B (Table 1B). In B, the electrophilicity at the C3 position was expected to be enhanced by the electron-withdrawing CO_2_Me group at C2, allowing the methyl group at C4 to undergo a 1,2-shift to C3 to yield 7′ (silyl-retained form) as shown by the pathway (a). Consistent with our assumption, the reaction in toluene at −40 °C to room temperature afforded 6-methyl-2,4-cyclohexadienone 7 (desilylated form) in 20% yield accompanied by the generation of 5-methyl phenol 10 in 80% yield as the main product (entry 1). The yield of 7 was not improved when other solvents, such as tetrahydrofuran (THF), acetonitrile (MeCN), methanol (MeOH) and 1,2-DCE, were used (entries 2–5). Instead, 4-hydroxy-4-methyl-2,5-cyclohexadienone 6 was obtained as the ring-opened and desilylated product, when THF, MeCN, or MeOH were used as solvents. Notably, the reaction in 1,2-DCE with MeOH (3.0 eq.) as an additive gave 6 in quantitative yield (entry 6), likely because MeOH works as a nucleophile toward the silyl moiety of B, facilitating desilylation (Table 1B, pathway (b)). The effects of other acids and solvents are described in Table S1. Given the generation of desilylated 7 instead of silyl-retained 7′, the formation of 7 is therefore most likely to have proceeded *via*6.

Optimization of the ring-opening reaction

**Table:** 

A. Ring-opening reaction^a^					
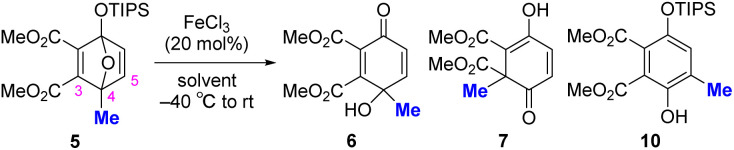					
Entry	Solvent	Time (h)	NMR yield (%)^b^		
			**6**	**7**	**10**
1	Tolune	24	0	20	80
2	THF	24	60	0	40
3	MeCN	30	79	Trace	7
4	MeOH	7	88 (81)^c^	0	Trace
5	1,2-DCE	1	0	11	21
6^d^	1,2-DCE	0.5	100	0	0

a Reactions were conducted on a 0.1 mmol scale.

b Determined by crude ^1^H NMR using 1,1,2,2-tetrachloroethane as an internal standard.

c Isolated yield.

d MeOH (3.0 eq.) was added as an additive.

**Table:** 

B. Proposed reaction mechanism
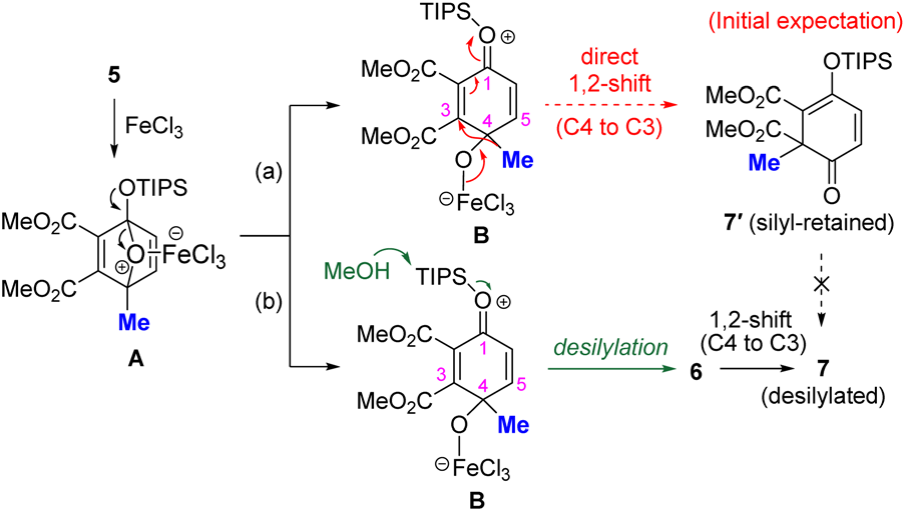

Encouraged by the formation of the desilylative ring-opened product 7, as outlined in Table 1, we investigated the FeCl_3_-catalyzed 1,2-shift of 6 to 7 (Table 2). The results obtained with other acids are presented in Table S2. The regioselective 1,2-shift of the methyl group at the C4 position of 6 to the C3 position proceeded smoothly in toluene under reflux conditions to afford 7 in 78% yield (entry 1). In contrast, THF and MeOH were ineffective solvents (entries 2 and 3). When MeCN was used as the solvent, 7 was obtained in 52% yield, along with recovered 6 in 19% yield (entry 4). Notably, when 1,2-DCE was used, 7 was obtained in 90% yield (entry 5). In these reactions, only a trace amount of phenol product 10′ was detected, which would otherwise arise from the competing 1,2-shift of the methyl group from C4 to C5. We assume that this selectivity arises because coordination of C1 carbonyl group or CO_2_Me groups to FeCl_3_ increases the electrophilicity at the C3 position, thereby promoting migration of the methyl group toward C3 position through electron donation from the phenolic hydroxy group at C4 position. Consequently, 6-methyl-2,4-cyclohexadienone 7, possessing a quaternary carbon center, was successfully constructed as the formal product resulting from a 1,2-shift of the C4 methyl group to the C3 position of 5 in a stepwise manner.

**Table 2:** Optimization of the 1,2-shift of 6 to 7^a^

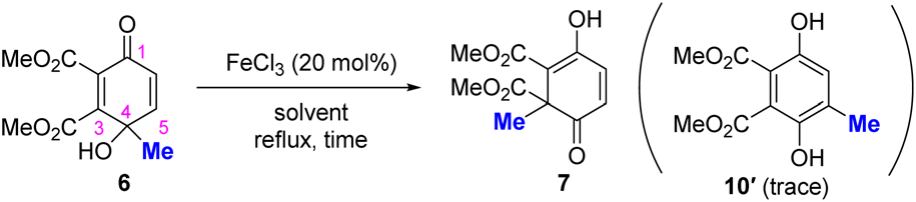				
Entry	Solvent	Time (h)	NMR yield (%)^b^	
			**7**	recov. **6**
1	Tolune	1	78	0
2	THF	24	Trace	89
3	MeOH	22	0	87
4	MeCN	144	52	19
5	1,2-DCE	0.5	90 (79)^c^	0

a Reactions were conducted on a 0.1 mmol scale.

b Determined by crude ^1^H NMR using 1,1,2,2-tetrachloroethane as an internal standard.

c Isolated yield.

Subsequently, we attempted to establish a one-pot protocol that would directly transform 5 into 7 without isolation of the ring-opened intermediate 6 (Table 3). Because both the formation of 6 from 5 and conversion of 6 to 7 are promoted by FeCl_3_, we envisioned that FeCl_3_ could sequentially trigger ring opening/desilylation and the subsequent 1,2-shift. Notably, the ring-opening step was promoted by the addition of MeOH (Table 1, entry 6). Considering these aspects, we investigated a one-pot reaction using an alcohol (ROH) as an additive. The influence of the amount of alcohol additive is described in Table S3. When MeOH was used as the alcohol, 7 was obtained in 60% yield, together with byproduct 15a, presumably arising from the nucleophilic addition of MeOH to intermediate 6 (entry 1). We evaluated other alcohols in an effort to suppress the formation of 15. Although EtOH did not improve the yield and still produced 15b (entry 2), *i*-PrOH effectively suppressed the nucleophilic addition, affording 7 in 78% yield (entry 3). In contrast, *t*-BuOH was ineffective (entry 4). These results indicate that tuning the steric bulkiness of the alcohol additive enables one-pot conversion of 5 to 7 by promoting O–Si bond cleavage and suppressing the undesired nucleophilic addition of alcohol to 6 to form 15.

**Table 3:** Investigation of the one-pot formation of 7 from 5^a^

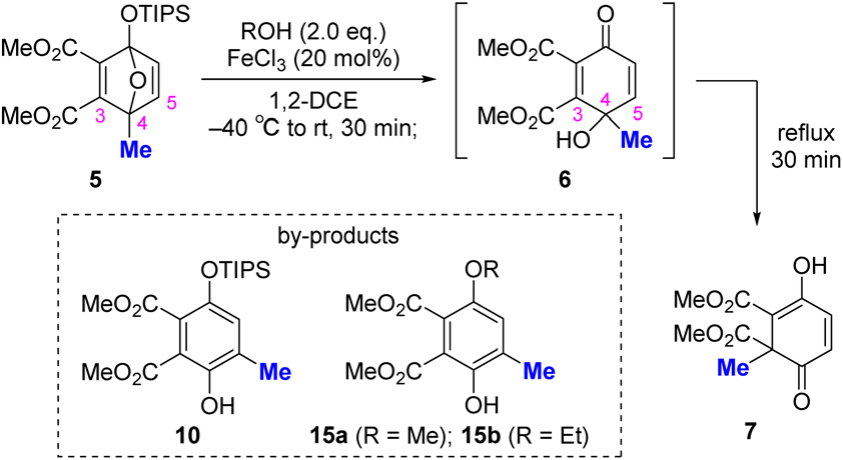				
Entry	ROH	NMR yield (%)^b^		
		**6**	**7**	**10**
1	MeOH	0	60	Trace
2	EtOH	0	61	Trace
3	*i*-PrOH	0	78 (78)^c^	Trace
4	*t*-BuOH	0	46	34

a Reactions were conducted on a 0.1 mmol scale.

b Determined by crude ^1^H NMR using 1,1,2,2-tetrachloroethane as an internal standard.

c Isolated yield.

**Scheme 2:**
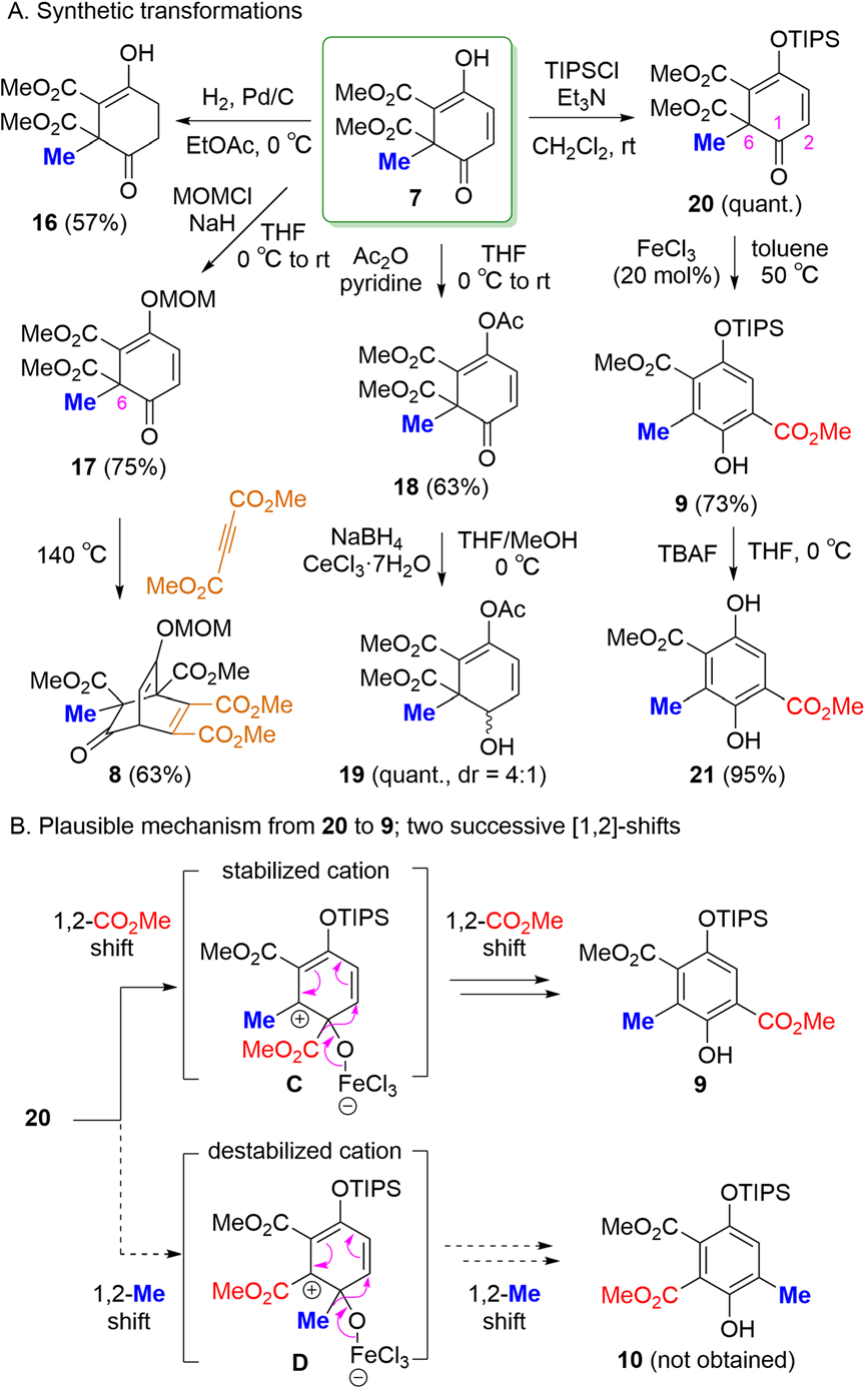
Further transformations of 7.

Further transformations of 7 were examined because 7 can serve as a useful reaction intermediate possessing olefin, enol, dienophile, α,β-unsaturated carbonyl moieties (Scheme 2A).^8,9^ Reduction of the olefin moiety of 7 over Pd/C under a hydrogen atmosphere afforded product 16. MOM ether 17, prepared by transformation of the enol moiety of 7 using methoxymethyl chloride (MOMCl), underwent a Diels–Alder reaction with dimethyl acetylenedicarboxylate upon heating to afford the fused-ring product 8. The structure of 8 was determined by X-ray analysis. The diastereoselectivity in the formation of 8 can be rationalized by the approach of dimethyl acetylenedicarboxylate from the side opposite to the bulky methoxycarbonyl group at the C6 position of 17, minimizing steric repulsion compared with approach from the side bearing the methyl group. This preference is also supported by DFT calculations (see SI for details). Acylation of the enol moiety of 7 with acetic anhydride (Ac_2_O) gave the acetylated product 18, whose α,β-unsaturated carbonyl moiety underwent Luche reduction to afford the corresponding alcohol 19 (dr = 4 : 1). The enol moiety of 7 was also converted into the corresponding triisopropylsilyl (TIPS) ether 20 using triisopropylsilyl chloride (TIPSCl).

Notably, treatment of 20 with catalytic FeCl_3_ in toluene at 50 °C afforded phenol 9. In this reaction, the methoxycarbonyl group at the C6 position of 20 migrated to C2. The structure of 20 was determined by X-ray structural analysis after desilylation using tetrabutylammonium fluoride (TBAF) to afford compound 21. In general, substituents that undergo migration are electron-rich groups, such as alkyl or aryl groups.^10^ However, electron-withdrawing groups may undergo this migration in specific systems.^10–12^ The proposed reaction mechanism involves two successive 1,2-shifts of the methoxycarbonyl group, as illustrated in Scheme 2B.^10^ The first 1,2-shift could generate either intermediate C or D. In intermediate C, the remaining methyl group can stabilize the positive charge through hyperconjugation. In contrast, the formation of intermediate D, involves interaction with the methoxycarbonyl group, which can destabilize the carbocation. Therefore, intermediate C is expected to be lower in energy than intermediate D, facilitating the migration of the methoxycarbonyl group to form C. A second 1,2-shift from C affords 9.^13^

## Conclusions

We established a desilylative ring-opening reaction of 5 to afford 6, followed by a CO_2_Me-induced regioselective 1,2-shift of the C4 methyl group to C3 to afford 7. Key features of this reaction include solvent tuning and the use of an alcohol additive to promote O–Si bond cleavage. Notably, 7 features a methyl-substituted quaternary carbon center that is difficult to access using existing methods but remains amenable to diverse transformations, underscoring its synthetic versatility. The present work expands the repertoire of skeletal rearrangements by revealing a new mode of methyl migration and demonstrates effective regioselective control over competing pathways.

## Conflicts of interest

There are no conflicts no declare.

## Supplementary Material

RA-016-D6RA01853J-s001

RA-016-D6RA01853J-s002

## Data Availability

CCDC 2520623, 2520617 and 2520616 contain the supplementary crystallographic data for this paper.^14*a*–14*c*^ The data supporting this article have been included as part of the supplementary information (SI). Supplementary information is available. See DOI: https://doi.org/10.1039/d6ra01853j.
