# Supplementary material for: Controlled formation of versatile methylated compounds based on ring opening of 4-methyl-1-siloxy-1,4-epoxy-1,4-dihydrobenzene
Source: RSC Adv. 2026 Mar 19;16(17):15586–90. doi: 10.1039/d6ra01853j (PMC13001159; doi:10.1039/d6ra01853j)

Supplementary Information (SI) for RSC Advances.
This journal is © The Royal Society of Chemistry 2026

Supplementary Information

Controlled Formation of Versatile Methylated Compounds Based on Ring Opening of 4-Methyl-1-Siloxy-1,4-Epoxy-1,4-Dihydrobenzene

Takaaki Aijima,[a] Jin Tokunaga,[a] Sota Yoshimura,[a] Yuki Itabashi,[b] Tsunayoshi Takehara,[c] Takeyuki Suzuki,[c] Shuji Akai,[a,c] and Yoshinari Sawama*[a]

[a]*Graduate School of Pharmaceutical Sciences, The University of Osaka, 1-6, Yamada-oka, Suita, Osaka 565-0871, Japan*

[b]*Institute for Open and Transdisciplinary Research Initiatives (OTRI), The University of Osaka, 1-6 Yamada-oka, Suita, Osaka 565-0871, Japan*

[c]*SANKEN, The University of Osaka, 8-1 Mihogaoka, Ibaraki, Osaka 567-0047, Japan*

E-mail: sawama@phs.osaka-u.ac.jp

Contents

1. General information.
2. Optimization of the reaction conditions.
3. Alternative reaction mechanism from 20 to 9.
4. Experimental procedures.
5. Scale-up reaction.
6. DFT calculations.
7. Data of X-ray crystallography.
8. References.
9. NMR spectra.

1. General information.

All reactions were carried out in dry solvents under argon atmosphere. Unless otherwise noted, all substrates and solvents were purchased from commercial sources and were used without further purification. **S2**[1)] was synthesized according to the reference. Flash column chromatography was performed with 40–50 μm Silica gel 60N (Kanto Chemical Co., Inc.). Gel permeation chromatography (GPC) was performed on JAI LaboAce LC-5060 Recycling Preparative HPLC with JAIGEL-2HR or JAIGEL-2H40. Melting points were measured on Yanaco MP-S3 and were uncorrected. IR spectra were recorded on SHIMADZU IRAffinity-1S as a thin film on NaCl. ^{1}H and ^{13}C NMR spectra were recorded on JEOL JNM-ECZL500 (^{1}H NMR: 500 MHz and ^{13}C NMR: 125 MHz), JEOL JNM-ECS400 (^{1}H NMR: 400 MHz and ^{13}C NMR: 100 MHz) or JEOL JNM-ECS300 (^{1}H NMR: 300 MHz and ^{13}C NMR: 75 MHz) with chemical shifts reported in δ (ppm) relative to the residual solvent signal for ^{1}H ($CDCl_3$: δ = 7.26 ppm, CD_3OD: δ = 3.31 ppm) and relative to the deuterated solvent signal for ^{13}C ($CDCl_3$: δ = 77.0 ppm). High resolution mass spectra were measured on JEOL JMS-T100LP.

2. Optimization of the reaction conditions.

Table S1. Optimization of the ring-opening reaction.[a]

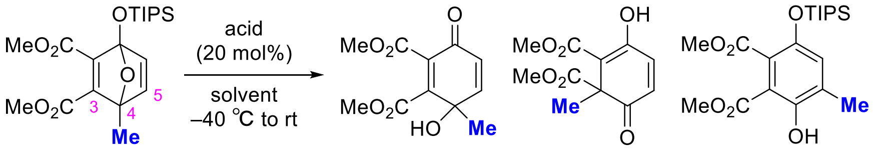

entry	acid	solvent	time (h)	NMR yield (%)[b]		
				**6**	**7**	**10**
1	$FeCl_3$	toluene	24	0	20	80
2	$BF_3 \cdot Et_2O$	toluene	0.33	21	0	78
3	$FeCl_3$	THF	24	60	0	40
4	$FeCl_3$	MeCN	30	79	trace	7
5	$FeCl_3$	MeOH	1	88 (81)[c]	0	trace
6	$BF_3 \cdot Et_2O$	MeOH	0.5	96	0	0
7	$FeCl_3$	1,2-DCE	1	0	11	21
8	$FeCl_3$	1,2-DCE/MeOH (9/1)	1	100 (94)[c]	0	0
9[d]	$FeCl_3$	1,2-DCE	0.5	100	0	0
10	$BF_3 \cdot Et_2O$	1,2-DCE/MeOH (9/1)	0.33	98	0	0
11	$TsOH \cdot H_2O$	1,2-DCE/MeOH (9/1)	0.5	95	0	0
12	$AlCl_3$	1,2-DCE/MeOH (9/1)	1	63	0	0
13	$TiCl_4$	1,2-DCE/MeOH (9/1)	0.5	88	0	0

[a]Reactions were conducted on a 0.1 mmol scale, [b]Determined by crude ^1H NMR using 1,1,2,2-tetrachloroethane as an internal standard.[c]Isolated yield. [d]MeOH (3.0 eq.) was added.

Table S2. Optimization of the 1,2-shift of **6** to **7**.[a]

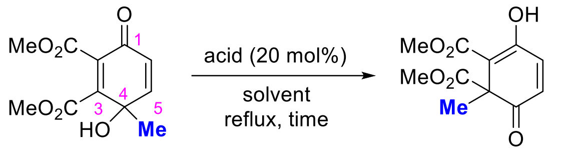

entry	acid	solvent	time (h)	NMR yield (%)[b]	
				**7**	recov. **6**
1	$FeCl_3$	toluene	1	78	0
2	$FeCl_3$	THF	24	trace	89
3	$FeCl_3$	MeOH	22	0	87
4	$FeCl_3$	MeCN	144	52	19
5	$FeCl_3$	1,2-DCE	0.5	90 (79)[c]	0
6	$BF_3 \cdot Et_2O$	1,2-DCE	0.5	98 (92)[c]	0
7	$TsOH \cdot H_2O$	1,2-DCE	3	90	0
8	$AlCl_3$	1,2-DCE	24	trace	96
9	$TiCl_4$	1,2-DCE	24	42	45
10	$FeCl_3$	1,2-DCE/MeOH (9/1)	21	13	50

[a]Reactions were conducted on a 0.1 mmol scale, [b]Determined by crude ^1H NMR using 1,1,2,2-tetrachloroethane as an internal standard.[c]Isolated yield.

Table S3. Investigation of the one-pot formation of **7** from **5**.[a]

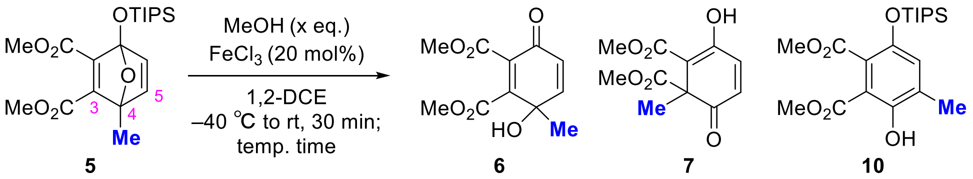

entry	ROH (eq.)	temp. (°C)	time (h)	NMR yield (%)[b]		
				**6**	**7**	**10**
1	MeOH (1.0)	–	–	8	52	24
2	MeOH (2.0)	–	–	75	12	0
3	MeOH (3.0)	–	–	quant.	0	0
4	MeOH (5.0)	–	–	quant.	0	0
5	MeOH (2.0)	rt	9	20	14	0
6	MeOH (2.0)	40	8	0	50	0
7	MeOH (2.0)	60	1	0	56	0
8	MeOH (2.0)	reflux	0.5	0	60	0
9	EtOH (2.0)	reflux	0.5	0	61	trace
10	*i*-PrOH (2.0)	reflux	0.5	0	78 (78)[c]	trace
11[d]	*i*-PrOH (2.0)	reflux	7	85	7	8
12	*t*-BuOH (2.0)	reflux	0.5	0	46	34
13	H_2O (2.0)	reflux	0.5	0	31	9

[a]Reactions were conducted on a 0.1 mmol scale, [b]Determined by crude ^1H NMR using 1,1,2,2-tetrachloroethane as an internal standard.[c]Isolated yield.
[d]$BF_3·Et_2O$ (20 mol%) was used instead of $FeCl_3$.

3. Alternative reaction mechanism from 20 to 9.

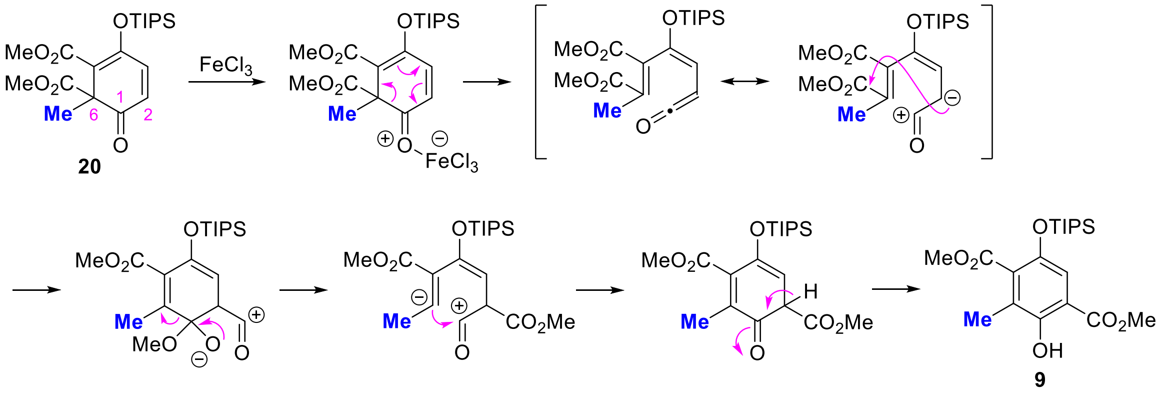

Lewis acid coordination to the 1 oxo group polarizes the cyclohexadienone and enables a 6π electrocyclic ring opening to generate a zwitterion with C1 exhibiting acylium character and C6/C2 carrying significant negative charge density by resonance. Charge delocalization selectively enhances nucleophilicity at C2,

which undergoes intramolecular nucleophilic acyl substitution onto the C6 attached ester through a compact 6-membered transition state. This step cleaves the C6–CO_2Me bond and transfers the acyl fragment to C2, producing the substitution pattern observed in the product. The resulting zwitterionic intermediate undergoes intramolecular nucleophilic attack of C6(–) onto the Lewis acid activated C1(+) to form the new C6–C1 σ bond and reform the ring. Subsequent deprotonation at C2 and collapse of the C1–LA complex (tautomerization) generates the aromatic ring and yields observed product **9**.

4. Experimental procedures.

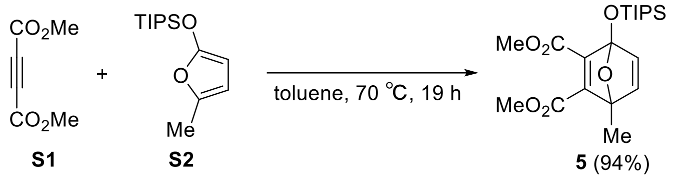

A mixture of **S1** (331 mg, 2.33 mmol) and **S2** (714 mg, 2.81 mmol) in toluene (2.8 mL) was heated at 70 °C for 19 h with stirring. The mixture was concentrated in vacuo, and the resulting residue was purified by flash column chromatography on silica gel (hexane/EtOAc = 10/1 with 5% Et_3N) to afford **5** (863 mg, 2.18 mmol) in 94 % yield.

Colorless oil; IR (NaCl) cm^{-1}: 2948, 2894, 2868, 1722, 1649, 1463, 1435, 1336, 1299, 1261, 1219, 1143, 1097, 997, 883, 831, 687; ^1H NMR (500 MHz, $CDCl_3$) δ: 6.92 (d, J = 5.1 Hz, 1H), 6.88 (d, J = 5.1 Hz, 1H), 3.81 (s, 3H), 3.74 (s, 3H), 1.80 (s, 3H), 1.19–1.10 (m, 3H), 1.07–1.03 (m, 18H); ^{13}C NMR (100 MHz, $CDCl_3$) δ: 164.7, 163.2, 157.4, 150.5, 147.9, 145.2, 111.8, 86.9, 52.1, 17.7, 16.0, 12.5; ESI-HRMS (m/z) calcd. for $C_{20}H_{32}NaO_6Si$ $[M+Na]^+$ 419.1860, found 419.1859.

Experimental procedure for Table 1:

$FeCl_3$ (3.2 mg, 0.020 mmol) was added to a solution of **5** (39.7 mg, 0.100 mmol) in a solvent (1.0 mL) at −40 °C. After being stirred at −40 °C for adequate time, water was added to the mixture. The resulting mixture was extracted with EtOAc. The combined organic layers were washed with brine, dried over Na_2SO_4, and concentrated in vacuo. The yields of each product were calculated by crude ^1H NMR using 1,1,2,2-tetrachloroethane (10.5 μL, 0.100 mmol) as an internal standard. The residue was purified by flash column chromatography on silica gel (hexane/EtOAc) to afford analytically pure **6**, **7** and **10**. For entry 4, the residue was purified by flash column chromatography on silica gel (hexane/EtOAc = 5/1 to 1/1) to afford **7** (19.4 mg, 0.0808 mmol) in 81% yield.

Experimental procedure for Table 2:

$FeCl_3$ (3.2 mg, 0.020 mmol) was added to a solution of **6** (24.0 mg, 0.100 mmol) in a solvent (1.0 mL) at room temperature. After being stirred under reflux conditions for adequate time, water was added to the mixture. The resulting mixture was extracted with EtOAc. The combined organic layers were washed with brine, dried over Na_2SO_4, and concentrated in vacuo. The yield was calculated by crude ^1H NMR using 1,1,2,2-tetrachloroethane (10.5 μL, 0.100 mmol) as an internal standard. For entry 5, the residue was purified

by flash column chromatography on silica gel (hexane/EtOAc = 5/1) to afford **7** (19.0 mg, 0.0791 mmol) in 79% yield.

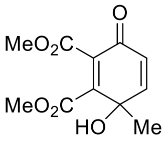

6

Pale yellow oil; IR (NaCl) cm^{-1}: 3474, 3008, 2957, 1739, 1667, 1642, 1436, 1392, 1331, 1283, 1263, 1088, 1054, 1020, 838, 800; ^{1}H NMR (500 MHz, $CDCl_3$) δ: 6.96 (d, *J* = 10.2 Hz, 1H), 6.26 (d, *J* = 10.2 Hz, 1H), 3.94 (s, 1H), 3.89 (s, 3H), 3.87 (s, 3H), 1.68 (s, 3H); ^{13}C NMR (125 MHz, $CDCl_3$) δ: 181.9, 165.3, 164.9, 152.8, 145.1, 135.5, 125.6, 68.4, 53.3, 52.8, 28.1; ESI-HRMS (m/z) calcd. for $C_{11}H_{12}NaO_6$ $[M+Na]^+$ 263.0526, found 263.0526.

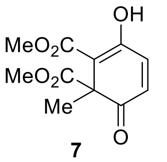

7

Yellow solid; m.p. 102–104 °C; IR (NaCl) cm^{-1}: 3071, 3008, 2959, 1756, 1679, 1631, 1592, 1436, 1412, 1371, 1331, 1253, 1224, 1149, 1108, 1051, 800; ^{1}H NMR (500 MHz, $CDCl_3$) δ: 12.11 (s, 1H), 7.04 (d, *J* = 10.1 Hz, 1H), 6.35 (d, *J* = 10.1 Hz, 1H), 3.81 (s, 3H), 3.67 (s, 3H), 1.60 (s, 3H); ^{13}C NMR (125 MHz, $CDCl_3$) δ: 195.9, 169.8, 169.8, 161.2, 140.3, 131.2, 106.5, 56.8, 52.9, 52.2, 23.7; ESI-HRMS (m/z) calcd. for $C_{11}H_{12}NaO_6$ $[M+Na]^+$ 263.0526, found 263.0530.

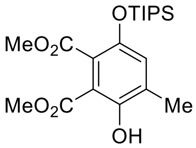

10

Colorless oil; IR (NaCl) cm^{-1}: 3172, 2947, 2868, 1739, 1679, 1591, 1442, 1348, 1301, 1236, 1211, 1162, 1079, 1026, 993, 933, 882, 797, 766, 685; ^{1}H NMR (500 MHz, $CDCl_3$) δ: 10.84 (s, 1H), 6.88 (s, 1H), 3.89 (s, 3H), 3.85 (s, 3H), 2.23 (d, *J* = 0.7 Hz, 3H), 1.30–1.19 (m, 3H), 1.08 (d, *J* = 7.3 Hz, 18H); ^{13}C NMR (125 MHz, $CDCl_3$) δ: 169.6, 167.9, 154.3, 144.4, 129.0, 127.5, 122.8, 108.4, 52.8, 52.1, 17.9, 16.2, 12.8; ESI-HRMS (m/z) calcd. for $C_{20}H_{32}NaO_6Si$ $[M+Na]^+$ 419.1860, found 419.1854.

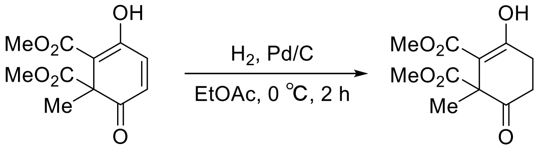

7 **16** (57%)

Pd/C (10%, 26.6 mg, 0.0250 mmol) was added to a solution of **7** (120 mg, 0.500 mmol) in EtOAc (5 mL) at room temperature and then hydrogen gas was purged. After being stirred at 0 °C for 2 h, the reaction mixture

was filtered through a membrane filter (Millipore, Omnipore™, 0.2 μm). The filtrate was concentrated in vacuo. The residue was purified by flash column chromatography on silica gel (hexane/EtOAc = 10/1) to afford **16** (69.4 mg, 0.287 mmol) in 57% yield.

White solid; m.p. 87–89 °C; IR (NaCl) cm^{-1}: 2997, 2955, 2847, 1756, 1714, 1662, 1615, 1442, 1349, 1316, 1251, 1217, 1184, 1156, 1100, 1068, 988, 834; ^1H NMR (500 MHz, $CDCl_3$) δ: 12.62 (s, 1H), 3.76 (s, 3H), 3.67 (s, 3H), 2.82–2.67 (m, 4H), 1.59 (s, 3H); ^{13}C NMR (125 MHz, $CDCl_3$) δ: 205.2, 171.6, 171.4, 171.1, 101.8, 56.2, 52.7, 51.9, 34.8, 28.4, 22.9; ESI-HRMS (m/z) calcd. for $C_{11}H_{14}NaO_6$ $[M+Na]^+$ 265.0683, found 265.0681.

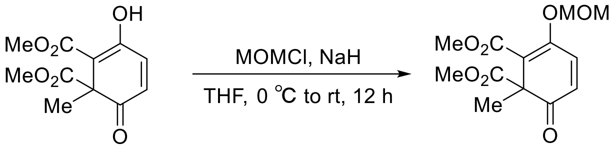

7 **17** (75%)

NaH (60%, dispersion in paraffin liquid, 90.0 mg, 2.25 mmol) was added to a solution of **7** (360 mg, 1.50 mmol) in THF (15 mL) at 0 °C. After being stirred at 0 °C for 30 min, MOMCl (171 μL, 2.25 mmol) was added to the mixture at 0 °C. After being stirred at 0 °C to room temperature for 12 h, saturated aqueous NH_4Cl solution was added to the mixture. The resulting mixture was extracted with EtOAc. The combined organic layers were washed with brine, dried over Na_2SO_4, and concentrated in vacuo. The residue was purified by flash column chromatography on silica gel (hexane/EtOAc = 3/1) to afford **17** (319 mg, 1.12 mmol) in 75% yield.

Yellow solid; m.p. 48–50 °C; IR (NaCl) cm^{-1}: 2997, 2954, 2834, 1760, 1729, 1679, 1637, 1436, 1319, 1220, 1155, 1086, 1045, 970, 927; ^1H NMR (500 MHz, CD_3OD) δ: 7.46 (d, J = 10.3 Hz, 1H), 6.29 (d, J = 10.3 Hz, 1H), 5.18 (s, 2H), 3.75 (s, 3H), 3.64 (s, 3H), 3.50 (s, 3H), 1.55 (s, 3H)); ^{13}C NMR (125 MHz, $CDCl_3$) δ: 196.0, 169.2, 164.3, 153.0, 139.5, 129.0, 121.3, 96.5, 59.2, 56.9, 52.9, 52.0, 23.2; ESI-HRMS (m/z) calcd. for $C_{13}H_{16}NaO_7$ $[M+Na]^+$ 307.0788, found 307.0788.

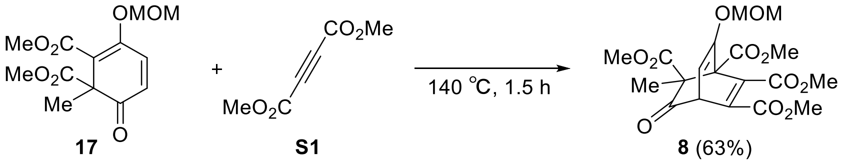

17 **S1** **8** (63%)

A mixture of **17** (71.1 mg, 0.250 mmol) in **S1** (0.50 mL, 4.1 mmol, 16 eq.) was heated at 140 °C for 1.5 h with stirring. After being cooled to room temperature, the mixture was directly purified by flash column chromatography on silica gel (Hexane/EtOAc = 3/1 with 1% Et_3N) to afford impure **8** (87.1 mg). The impure **8** was further purified by GPC to afford **8** (67 mg, 0.157 mmol) in 63% yield.

White solid; m.p. 130–133 °C; IR (NaCl) cm^{-1}: 3003, 2955, 2905, 2847, 1753, 1725, 1650, 1622, 1436, 1327, 1298, 1254, 1223, 1202, 1155, 1097, 1077, 990, 736; ^1H NMR (500 MHz, $CDCl_3$) δ: 5.50 (d, J = 7.0 Hz, 1H), 5.08 (d, J = 5.9 Hz, 1H), 4.96 (d, J = 5.9 Hz, 1H), 4.45 (d, J = 7.0 Hz, 1H), 3.82 (s, 3H), 3.80 (s, 3H), 3.79 (s, 3H), 3.73 (s, 3H), 3.44 (s, 3H), 1.56 (s, 3H); ^{13}C NMR (125 MHz, $CDCl_3$) δ: 196.8, 169.2, 166.1, 166.0,

162.5, 160.3, 145.1, 137.6, 96.3, 94.3, 64.9, 56.7, 53.2, 53.1, 52.9, 52.8, 52.7, 52.6, 21.6; ESI-HRMS (m/z) calcd. for $C_{19}H_{22}NaO_{11}$ $[M+Na]^+$ 449.1054, found 449.1053.

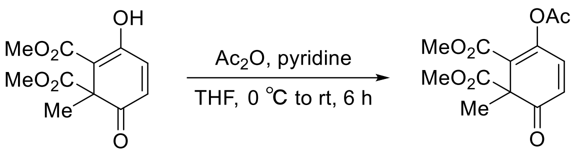

7 **18** (63%)

Ac_2O (1.89 mL, 20.0 mmol) was added to a solution of **7** (480 mg, 2.00 mmol) and pyridine (4.83 mL, 60.0 mmol) in THF (20 mL) at 0 ºC. After being stirred at room temperature for 6 h, water was added to the mixture. The resulting mixture was extracted with EtOAc. The combined organic layers were washed with brine, dried over Na_2SO_4, and concentrated in vacuo. The residue was purified by flash column chromatography on silica gel (hexane/EtOAc = 5/1 to 3/1) to afford **18** (354 mg, 1.25 mmol) in 63% yield and recovered **8** (161 mg, 0.670 mmol) in 34% yield. The yield of **18** based on recovered starting material is 95%.

Pale yellow solid; m.p. 101–103 ºC; IR (NaCl) cm^{-1}: 3065, 3004, 2956, 1761, 1726, 1679, 1436, 1401, 1371, 1315, 1282, 1228, 1179, 1046, 1030, 861; ^1H NMR (500 MHz, $CDCl_3$) δ: 6.90 (d, J = 10.1 Hz, 1H), 6.31 (d, J = 10.1 Hz, 1H), 3.77 (s, 3H), 3.69 (s, 3H), 2.30 (s, 3H), 1.67 (s, 3H); ^{13}C NMR (125 MHz, $CDCl_3$) δ: 195.4, 168.5, 168.2, 162.7, 148.0, 141.1, 129.1, 126.9, 59.4, 53.1, 52.3, 23.3, 20.7; ESI-HRMS (m/z) calcd. for $C_{13}H_{14}NaO_7$ $[M+Na]^+$ 305.0632, found 305.0628.

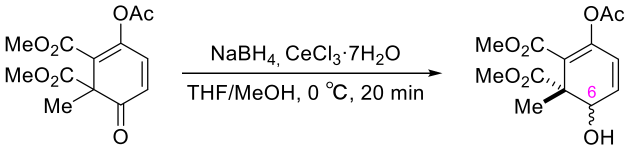

18 **19** (quant., dr = 4:1)

$CeCl_3 \cdot 7H_2O$ (186 mg, 0.500 mmol) and $NaBH_4$ (18.9 mg, 0.500 mmol) were successively added to a solution of **18** (141 mg, 0.500 mmol) in MeOH/THF (1/1, 5 mL) at 0 ºC. After being stirred at 0 ºC for 20 min, water was added to the mixture. The resulting mixture was extracted with $CHCl_3$. The combined organic layers were washed with brine, dried over Na_2SO_4, and concentrated in vacuo. The residue was purified by flash column chromatography on silica gel (hexane/EtOAc = 1/1) to afford impure **19-major** and impure **19-minor**. The impure **19-major** and **19-minor** were further purified by GPC to afford analytically pure sample. The yields of each diastereomers were calculated by crude ^1H NMR using 1,1,2,2-tetrachloroethane as an internal standard. The relative stereochemistry at C6 was determined by NOE correlations (see page S36).

19-major

Colorless oil; IR (NaCl) cm^{-1}: 3495, 3001, 2954, 2848, 1753, 1723, 1589, 1436, 1371, 1310, 1274, 1225, 1179, 1104, 1040, 1015, 900, 760; ^1H NMR (500 MHz, $CDCl_3$) δ: 6.04 (dd, J = 10.0, 2.3 Hz, 1H), 5.76 (dd, J = 10.0, 2.8 Hz, 1H), 5.06–5.03 (m, 1H), 3.72 (s, 3H), 3.71 (s, 3H), 2.21 (s, 3H), 2.01 (d, J = 5.6 Hz, 1H), 1.50 (s, 3H); ^{13}C NMR (125 MHz, $CDCl_3$) δ: 175.6, 168.2, 165.1, 150.0, 137.4, 123.1, 119.6, 73.0, 53.5, 52.7, 51.8, 20.7, 12.3; ESI-HRMS (m/z) calcd. for $C_{13}H_{16}NaO_7$ $[M+Na]^+$ 307.0788, found 307.0788.

19-minor

Colorless oil; IR (NaCl) cm^{-1}: 3491, 3000, 2953, 2851, 1726, 1436, 1371, 1268, 1232, 1185, 1135, 1071, 1040, 763; ^1H NMR (300 MHz, $CDCl_3$) δ: 6.29 (dd, J = 9.8, 5.0 Hz, 1H), 5.94 (d, J = 9.8 Hz, 1H), 4.04 (d, J = 5.0 Hz, 1H), 3.74 (s, 3H), 3.72 (s, 3H), 2.32 (s, 1H), 2.22 (s, 3H), 1.54 (s, 3H); ^{13}C NMR (125 MHz, $CDCl_3$) δ: 173.1, 168.7, 165.5, 147.5, 131.4, 124.6, 119.4, 70.4, 52.5, 52.4, 51.8, 20.7, 20.6; ESI-HRMS (m/z) calcd. for $C_{13}H_{16}NaO_7$ $[M+Na]^+$ 307.0788, found 305.0789.

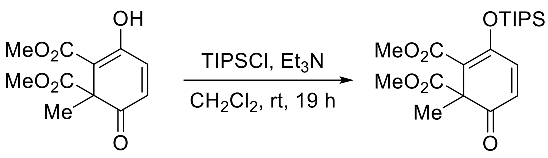

7 **20** (quant., NMR yield)

Et_3N (27.7 μL, 0.200 mmol) was added to a solution of **7** (24.0 mg, 0.100 mmol) in CH_2Cl_2 (1.0 mL) at room temperature. After being stirred at room temperature for 10 min, TIPSCl (42.4 μL, 0.200 mmol) was added to the mixture at room temperature. After being stirred at room temperature for 19 h, saturated aqueous $NaHCO_3$ solution was added to the mixture. The resulting mixture was extracted with $CHCl_3$. The combined organic layers were washed with brine, dried over Na_2SO_4, and concentrated in vacuo. The residue was purified by flash column chromatography on silica gel (hexane/EtOAc = 10/1 with 1% Et_3N) to afford impure **20** containing TIPSOH. The impure **20** was further purified by GPC to obtain analytically pure sample. The yield was calculated by crude ^1H NMR using 1,1,2,2-tetrachloroethane as an internal standard.

Yellow oil; IR (NaCl) cm^{-1}: 2949, 2894, 2869, 1761, 1729, 1693, 1679, 1634, 1567, 1455, 1436, 1408, 1328, 1207, 1110, 1081, 1053, 1030, 883, 820, 794, 782, 688; ^1H NMR (500 MHz, $CDCl_3$) δ: 6.90 (d, J = 10.2 Hz, 1H), 6.25 (d, J = 10.2 Hz, 1H), 3.74 (s, 3H), 3.67 (s, 3H), 1.60 (s, 3H), 1.30–1.21 (m, 3H), 1.13 (d, J = 7.2 Hz, 18H); ^{13}C NMR (125 MHz, $CDCl_3$) δ: 196.4, 169.7, 164.9, 151.5, 143.6, 128.9, 118.5, 59.0, 52.7, 51.5, 23.2, 17.7, 17.6, 13.2; ESI-HRMS (m/z) calcd. for $C_{20}H_{32}NaO_6Si$ $[M+Na]^+$ 419.1860, found 419.1859.

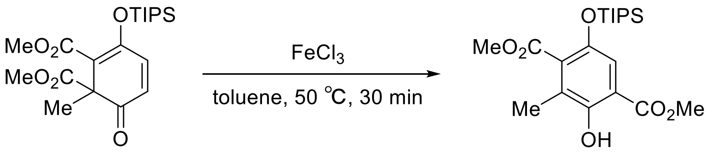

20 **9** (73%)

$FeCl_3$ (6.1 mg, 0.038 mmol) was added to a solution of **20** (74.1 mg, 0.187 mmol) in toluene (1.9 mL) at room temperature. After being stirred at 50 °C for 30 min, water was added to the mixture. The resulting mixture was extracted with EtOAc. The combined organic layers were washed with brine, dried over Na_2SO_4, and concentrated in vacuo. The residue was purified by flash column chromatography on silica gel (hexane/EtOAc = 50/1 with 1% Et_3N) to afford **9** (53.8 mg, 0.136 mmol) in 73% yield.

Colorless oil; IR (NaCl) cm^{-1}: 3204, 2949, 2893, 2868, 1739, 1679, 1615, 1468, 1441, 1386, 1349, 1271, 1247, 1214, 1140, 1057, 881, 812, 791, 728, 684; ^1H NMR (500 MHz, $CDCl_3$) δ: 10.69 (s, 1H), 7.14 (d, J = 0.4 Hz, 1H), 3.95 (s, 3H), 3.89 (s, 3H), 2.16 (d, J = 0.4 Hz, 3H), 1.29–1.21 (m, 3H), 1.08 (d, J = 7.4 Hz, 18H); ^{13}C NMR (125 MHz, $CDCl_3$) δ: 170.3, 167.8, 154.1, 143.8, 133.7, 124.9, 114.9, 111.5, 52.6, 52.1, 17.9, 12.7, 12.7; ESI-HRMS (m/z) calcd. for $C_{20}H_{32}NaO_6Si$ $[M+Na]^+$ 419.1860, found 419.1856.

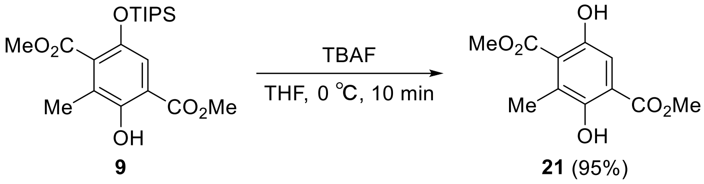

TBAF (1.0 M THF solution, 0.35 mL, 0.35 mmol) was added to a solution of **9** (39.7 mg, 0.100 mmol) in THF (1.0 mL) at 0 °C. After being stirred at 0 °C for 10 min, water was added to the mixture. The resulting mixture was extracted with EtOAc. The combined organic layers were washed with brine, dried over Na_2SO_4, and concentrated in vacuo. The residue was purified by flash column chromatography on silica gel (hexane/EtOAc = 10/1) to afford **21** (22.8 mg, 0.0949 mmol) in 95% yield.

Yellow solid; m.p. 152–154 °C; IR (NaCl) cm^{-1}: 3003, 2955, 2905, 2847, 1753, 1725, 1650, 1622, 1436, 1327, 1298, 1254, 1223, 1202, 1155, 1097, 1077, 990, 736; ^1H NMR (500 MHz, $CDCl_3$) δ: 10.57 (s, 1H), 9.79 (s, 1H), 7.35 (d, J = 0.7 Hz, 1H), 4.00 (s, 3H), 3.96 (s, 3H), 2.46 (d, J = 0.7 Hz, 3H); ^{13}C NMR (125 MHz, $CDCl_3$) δ: 170.8, 170.1, 152.1, 152.0, 128.6, 119.7, 116.0, 114.5, 52.7, 52.6, 14.1; ESI-HRMS (m/z) calcd. for $C_{11}H_{12}NaO_6$ $[M+Na]^+$ 263.0526, found 263.0536.

5. Scale-up reaction.

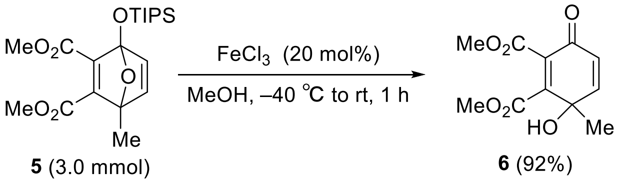

$FeCl_3$ (97.3 mg, 0.600 mmol) was added to a solution of **5** (1.19 g, 3.00 mmol) in MeOH (30 mL) at −40 °C. After being stirred at room temperature for 1 h, water was added to the mixture. The resulting mixture was extracted with EtOAc. The combined organic layers were washed with brine, dried over Na_2SO_4, and concentrated in vacuo. The residue was purified by flash column chromatography on silica gel (hexane/EtOAc = 2/1) to afford **6** (666 mg, 2. 77 mmol) in 92% yield.

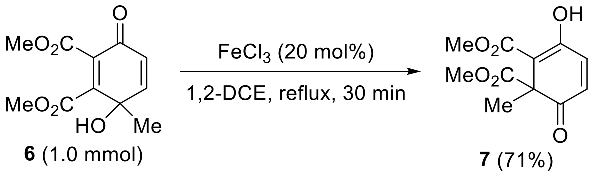

$FeCl_3$ (32.4 mg, 0.200 mmol) was added to a solution of **6** (240 mg, 1.00 mmol) in 1,2-DCE (10 mL) at room temperature. After being stirred under reflux conditions for 30 min, water was added to the mixture. The resulting mixture was extracted with EtOAc. The combined organic layers were washed with brine, dried over Na_2SO_4, and concentrated in vacuo. The residue was purified by flash column chromatography on silica gel (hexane/EtOAc = 5/1 to 1/1) to afford **7** (171 mg, 0.712 mmol) in 71% yield.

6. DFT calculations.

Theoretical Calculations

The density functional theory (DFT) calculations were performed by Gaussian 16 (Revision C.02; Gaussian Inc., Wallingford, CT, USA). The functional and basis set were M06–2X and 6–311+G (d, p). Optimized ground-state geometries were examined by frequency analysis to possess no negative frequency. Optimized transition states geometries were examined by frequency analysis to possess only one imaginary frequency. For each transition states, intrinsic reaction coordinate (IRC) analysis was performed to ensure that it connects the reactant and product.

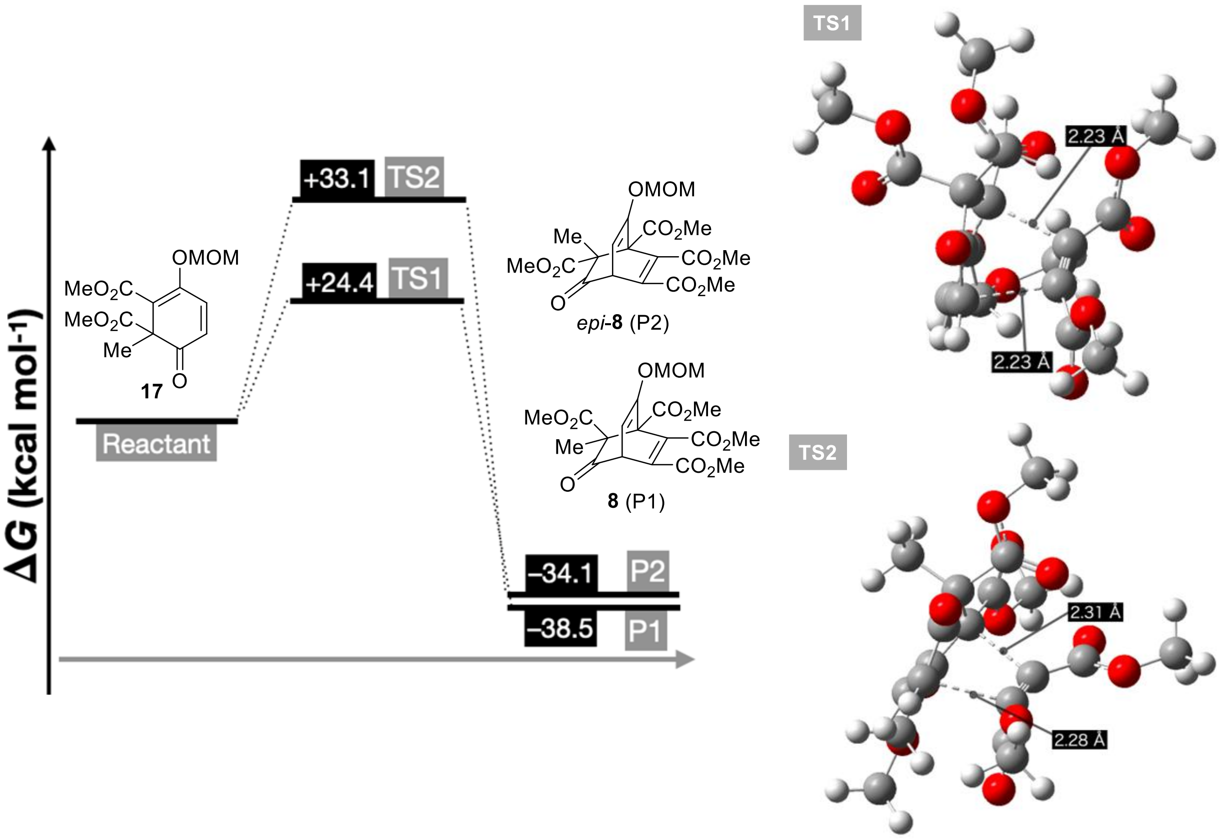

Figure S1. DFT calculation of Diels-Alder reaction between **17** and dimethyl acetylenedicarboxylate (**S1**).

MOM ether **17** underwent a Diels–Alder reaction with **S1** upon heating to afford the fused ring **8**, while no formation of *epi*-**8** was observed. Therefore, DFT calculations were performed to clarify the origin of the observed stereoselectivity. The possible pathways for the Diels–Alder reaction were identified via the transition structures **TS1** and **TS2**, with activation energies of +24.4 and +33.1 kcal mol^{-1}, respectively. Thus, the reaction was predicted to proceed preferentially through **TS1**, which is consistent with the exclusive formation of **8**.

The Cartesian coordinates

Substrates

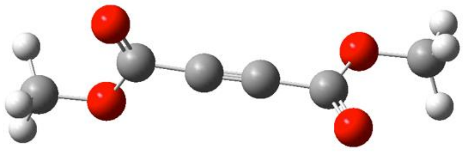

–532.9441 Hartree

Atom	X	Y	Z
C	-0.59621733	-0.20416017	0.06565117
C	0.59621764	-0.20415224	-0.06566092
C	-2.03674015	-0.24896857	0.27996637
C	2.03673896	-0.24894280	-0.27998563
O	2.56611991	-0.96092076	-1.08478734
O	-2.56612324	-0.96100995	1.08471054
O	2.66695809	0.59804304	0.53054661
O	-2.66695580	0.59808206	-0.53050084
C	4.09317342	0.62032010	0.39224205
H	4.44217599	1.35166009	1.11499965
H	4.50513337	-0.36556223	0.60662744
H	4.36637998	0.91371022	-0.62114396
C	-4.09317206	0.62035679	-0.39219555
H	-4.44217028	1.35174935	-1.11490181
H	-4.50513598	-0.36550875	-0.60664905
H	-4.36637758	0.91367750	0.62121108

SM MOM-protected diene

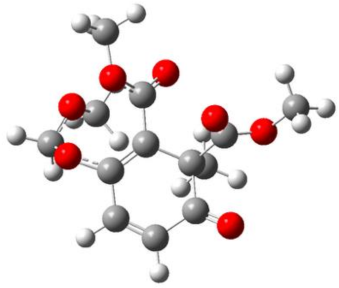

–1031.1979 Hartree

Atom	X	Y	Z
C	-1.70619121	-1.84739291	-0.22829781
C	-0.72813040	-2.90955175	-0.53697980
C	0.56007504	-2.60281129	-0.73629994
C	1.05837228	-1.22863982	-0.65227622
C	0.25869107	-0.22221490	-0.26901040
C	-1.12940579	-0.50883980	0.26172256
H	-1.12040604	-3.91011400	-0.67225277
H	1.28319528	-3.35871424	-1.02484136
C	-2.08272417	0.59287670	-0.19958316
O	-2.89694484	-2.03544094	-0.28240727
O	2.36527088	-1.06577449	-1.02435352
C	-1.08154855	-0.64977061	1.80558216
H	-0.70147163	0.27341417	2.24126254
H	-2.08473737	-0.84820898	2.18155988
H	-0.42606294	-1.48166560	2.07413901
O	-2.00018503	1.11265577	-1.27933510
O	-3.01217878	0.88531779	0.70282461
C	-3.97385621	1.85979195	0.28873084
H	-4.65224190	1.97806864	1.12890298
H	-3.47528216	2.80063775	0.05632840
H	-4.50707447	1.50421613	-0.59270483
C	0.70735503	1.19965286	-0.18819720
O	0.39714678	1.91978277	0.73104283
O	1.43128938	1.58003272	-1.22786579
C	1.99131378	2.88982305	-1.12865321
H	2.53116823	3.04818474	-2.05803013
H	1.20265231	3.63203588	-1.00746373
H	2.67043927	2.93002092	-0.27575723

C	3.28877636	-0.91630125	0.03957004
H	3.22348101	-1.78416186	0.71069595
H	4.25969796	-0.88009386	-0.45205737
C	2.68905577	0.10659586	2.10518837
H	2.39916702	1.09356080	2.46037553
H	1.82930814	-0.56522104	2.19030778
H	3.51625096	-0.27629539	2.71081784
O	3.08325391	0.26155481	0.75006173

Product MOM–DA–1 (more thermodynamically stable)

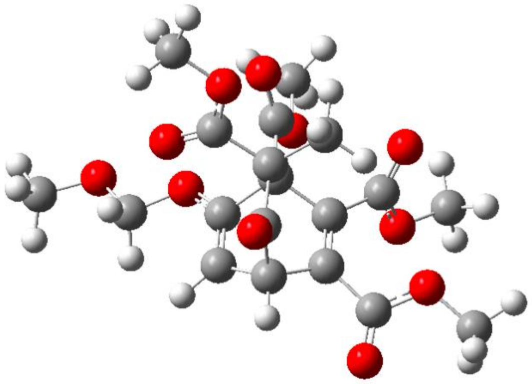

–1564.2034 Hartree

Atom	X	Y	Z
C	-0.01682789	2.25117671	0.58631897
C	0.59572033	1.36343613	1.69218663
C	-0.54377043	0.43490867	2.06154870
C	-0.94797880	-0.32233784	1.04077298
C	-0.21128556	-0.07409008	-0.27946900
C	-0.45331267	1.43400087	-0.65341861
H	-1.00098035	0.48148377	3.03811778
C	-1.95419913	1.67891932	-0.86133109
O	-0.14957317	3.43792722	0.66808485
O	-1.93839332	-1.22085498	0.96868287
C	0.38062904	1.89554205	-1.85504024
H	0.08093843	1.35242189	-2.75047016
H	0.21715431	2.96056031	-2.01274708
H	1.44239520	1.72282607	-1.67717910
O	-2.81179939	1.29125439	-0.11198020
O	-2.20151767	2.40351514	-1.94632234
C	-3.58553189	2.64119993	-2.21386192
H	-3.60981085	3.23643775	-3.12235346
H	-4.10092127	1.69153210	-2.35793420
H	-4.04196262	3.18061757	-1.38410205
C	-0.72905891	-1.01367232	-1.35892534
O	-1.45948325	-0.68220380	-2.24942134
O	-0.28698944	-2.25260141	-1.15889458
C	-0.75966331	-3.22881345	-2.09201903
H	-0.32534075	-4.17282220	-1.77498950
H	-1.84792189	-3.27393901	-2.05830309

H	-0.43240082	-2.96726651	-3.09859290
H	0.95965310	1.97349991	2.51365457
C	1.28010767	-0.20741696	0.01788132
C	1.69502178	0.58004366	1.01176809
C	3.10062752	0.78396530	1.45383447
C	2.14330469	-1.03698711	-0.87579140
O	3.40019105	1.17566862	2.54967122
O	2.18789543	-0.87632355	-2.06609069
O	2.81199127	-1.97365599	-0.21032063
O	3.98609495	0.51394906	0.48892037
C	3.69070945	-2.76754363	-1.01026570
H	4.44887537	-2.13374568	-1.47207091
H	4.14526326	-3.48135223	-0.32904484
H	3.12881993	-3.28076769	-1.79099021
C	5.35965926	0.64140608	0.86610778
H	5.56513625	1.65856783	1.19911783
H	5.59282137	-0.05468554	1.67220826
H	5.93254930	0.40614892	-0.02662833
C	-2.88425481	-1.15305669	2.00622708
H	-3.31321580	-0.14004014	2.02074234
H	-2.39872242	-1.38027251	2.96913473
O	-3.83362784	-2.11251651	1.69753745
C	-4.90813842	-2.08895688	2.60970230
H	-5.42246678	-1.12102167	2.58967334
H	-5.60135378	-2.86948124	2.30396823
H	-4.56736571	-2.29230980	3.63249711

Product MOM–DA–2

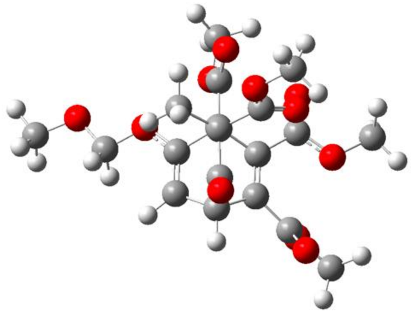

–1564.1964 Hartree

Atom	X	Y	Z
C	-0.17276390	0.44374513	-2.22235093
C	-0.19166437	-1.07567038	-1.91412023
C	1.22323427	-1.39073771	-1.48360673
C	1.60312689	-0.70248718	-0.40400763
C	0.56202421	0.26938034	0.15352349
C	0.32911970	1.28955834	-1.01996478
H	1.84697674	-2.04845095	-2.07004026
C	-0.77746395	2.28073565	-0.64328607
O	-0.45353142	0.91387940	-3.28448569
O	2.81833307	-0.63850811	0.16172921
C	1.60821344	2.01860056	-1.45846762
H	2.40007780	1.30122413	-1.67790538
H	1.39731393	2.58920001	-2.36145837
H	1.94172535	2.69761233	-0.67572559
O	-1.73279862	2.01268404	0.03694993
O	-0.58153345	3.47474341	-1.19059823
C	-1.58804781	4.44934322	-0.90113916
H	-1.27699982	5.35251399	-1.41847046
H	-2.55616824	4.10712190	-1.26625352
H	-1.64326470	4.61634308	0.17436388
C	1.02466761	0.97308681	1.42968167
O	1.00876427	2.16584811	1.56978627
O	1.42991890	0.10172947	2.33850173
C	1.69082365	0.64983141	3.63168669
H	2.01995419	-0.18525525	4.24361042
H	2.46696434	1.41213637	3.56860408
H	0.77118801	1.08314734	4.02602137

H	-0.52922090	-1.61963178	-2.79194781
C	-0.75971386	-0.49594602	0.33855730
C	-1.12123070	-1.20672686	-0.73146082
C	-2.38168300	-1.98098523	-0.92352182
C	-1.64503815	-0.22924213	1.51485862
O	-3.15188391	-1.76830052	-1.81910098
O	-1.28707878	0.24752179	2.55781789
O	-2.91536700	-0.54466472	1.25108533
O	-2.49901379	-2.97802838	-0.04443921
C	-3.84943797	-0.20380698	2.27592168
H	-3.80862627	0.86845523	2.46815532
H	-4.82493601	-0.48773214	1.89008148
H	-3.62176951	-0.74630038	3.19379506
C	-3.72494226	-3.70759181	-0.12402244
H	-4.56519173	-3.03887197	0.06814089
H	-3.84073412	-4.15312331	-1.11203402
H	-3.66119319	-4.47592882	0.64146193
C	3.81212043	-1.42037629	-0.44602436
H	3.51769055	-2.48184211	-0.41383587
H	3.93935510	-1.10532462	-1.49572126
O	4.96015033	-1.19262103	0.28926260
C	6.05778423	-1.91681720	-0.22088884
H	6.28148508	-1.62358252	-1.25364912
H	6.91209079	-1.68250351	0.40992482
H	5.87083523	-2.99683667	-0.18739656

TS MOM−1 (more thermodynamically stable)

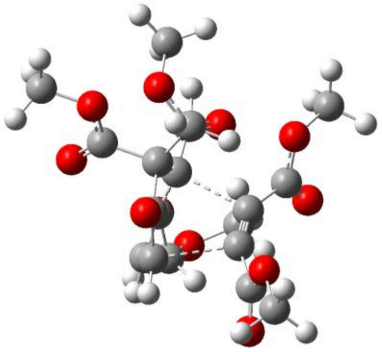

−1564.1031 Hartree (i = −348.87)

Atom	X	Y	Z
C	-1.08559608	1.87008451	-0.57798928
C	-1.46771738	0.92112949	-1.64443794
C	-0.54979976	0.07350670	-2.21381372
C	0.55203420	-0.34862074	-1.44841447
C	0.70839011	0.15821494	-0.15514373
C	0.24261903	1.57788788	0.14622719
H	-0.77418601	-0.40511357	-3.15864562
C	1.24531681	2.54567256	-0.53481282
O	-1.74735200	2.84405875	-0.31501760
O	1.37592820	-1.32816826	-1.84498092
C	0.06610607	1.88803434	1.63716378
H	1.01701586	1.82026785	2.16263054
H	-0.32410923	2.89768549	1.74175085
H	-0.64232989	1.18822044	2.07796328
O	1.70643782	2.37567855	-1.62904208
O	1.49036388	3.61585169	0.21592350
C	2.35515518	4.58747733	-0.38012031
H	2.44779703	5.38491689	0.35180388
H	3.32619496	4.14147127	-0.59578570
H	1.91774949	4.96062336	-1.30589868
C	1.92316325	-0.32933678	0.58891266
O	2.14540719	-1.46699794	0.90151611
O	2.75998620	0.68205119	0.85223051
C	3.96298287	0.32164686	1.53811309
H	4.51983845	1.24653249	1.65971536
H	3.72362021	-0.11598919	2.50782791
H	4.52910915	-0.39740311	0.94682414

H	-2.39915154	1.14885199	-2.15134767
C	-1.09358404	-0.88027044	0.64279475
C	-2.10852225	-0.41819085	0.10718513
C	-3.54156951	-0.28169914	-0.12252965
C	-0.51619149	-1.87828965	1.55228118
O	-4.13748725	-0.79401063	-1.03196958
O	-0.49150133	-3.05242024	1.31308241
O	-0.05662168	-1.31244343	2.66946053
O	-4.09199538	0.51180985	0.79757089
C	0.61075433	-2.21071162	3.56387057
H	1.47068965	-2.65059036	3.05837236
H	0.92200709	-1.60233596	4.40883897
H	-0.07124009	-2.99703158	3.88629825
C	-5.48948634	0.76814081	0.62165087
H	-5.66400254	1.24416095	-0.34327482
H	-6.05271831	-0.16349467	0.67281132
H	-5.76619090	1.43363458	1.43406255
C	0.86079840	-2.36430088	-2.70479683
H	0.97577770	-2.05691426	-3.74557956
H	-0.19657719	-2.51924020	-2.44309243
O	1.60324971	-3.49203911	-2.51792405
C	1.40874626	-4.08318471	-1.23353676
H	0.34868219	-4.30671371	-1.07490670
H	1.98511020	-5.00559450	-1.23047346
H	1.75399289	-3.41605133	-0.44231481

TS MOM–2

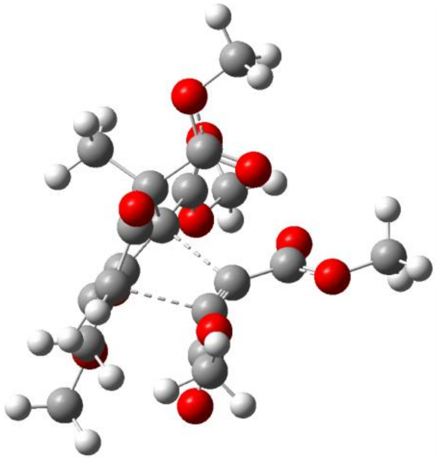

–1564.0893 Hartree (i = –348.70)

Atom	X	Y	Z
C	1.16158866	0.61510869	-1.81989207
C	0.21492274	1.74004176	-1.62263643
C	-1.12909410	1.52527804	-1.44562207
C	-1.57182256	0.30881796	-0.88533070
C	-0.62451736	-0.67888177	-0.58956193
C	0.59104720	-0.76641638	-1.51314532
H	-1.80651916	2.36176910	-1.55193137
C	1.72524516	-1.60890836	-0.90459752
O	2.26365215	0.77195333	-2.28364245
O	-2.85536455	0.12262351	-0.53276784
C	0.14045600	-1.33556107	-2.88370529
H	-0.63752670	-0.70023210	-3.30964897
H	0.99207321	-1.36853701	-3.56140336
H	-0.24634826	-2.34474667	-2.74601558
O	2.13778284	-1.48127940	0.21672238
O	2.24527019	-2.45840850	-1.78264527
C	3.29886714	-3.27961082	-1.27527472
H	3.60696512	-3.90798159	-2.10639756
H	4.12570301	-2.65912086	-0.93036742
H	2.92645382	-3.88465872	-0.44835819
C	-1.02488202	-2.01376504	-0.01802733
O	-0.37077256	-3.00676970	-0.23240731
O	-2.13258849	-1.99835160	0.70024253
C	-2.41667709	-3.21003959	1.39920735
H	-3.34490079	-3.02513080	1.93269336

H	-2.53101587	-4.03694002	0.69829057
H	-1.60370466	-3.42293575	2.09388683
H	0.58441400	2.71184669	-1.93351644
C	0.49936830	0.52143702	1.02993438
C	0.83464182	1.61671716	0.57038062
C	1.35405359	2.97598917	0.58742814
C	0.64646222	-0.41684301	2.16045862
O	0.70437516	3.93925983	0.89964742
O	-0.05639817	-1.35509050	2.40983231
O	1.69608053	-0.05112629	2.89580999
O	2.62367972	3.01126867	0.18612678
C	2.01871352	-0.94862454	3.96137295
H	2.23087412	-1.93818288	3.55584406
H	2.90051743	-0.52890826	4.43730881
H	1.19087549	-1.01390992	4.66756183
C	3.21191615	4.31389600	0.13238182
H	2.65223668	4.95294230	-0.55136731
H	3.21692443	4.76639535	1.12394613
H	4.22470749	4.15887974	-0.22768357
C	-3.71071492	1.23544241	-0.48316926
H	-3.88167446	1.63420599	-1.49660833
H	-3.27171525	2.01816560	0.15611472
O	-4.89040123	0.75320889	0.05263896
C	-5.86356338	1.76841976	0.16179457
H	-6.11185355	2.18857361	-0.82049968
H	-6.75233826	1.30962570	0.58869577
H	-5.52273931	2.57559002	0.82102104

7. Data of X-ray crystallography.

Compound **7**; CCDC Deposition Number 2520623

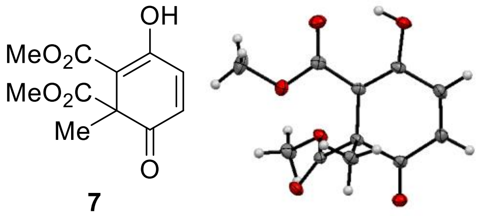

7

checkCIF/PLATON report

Structure factors have been supplied for datablock(s) jt-me3-1

THIS REPORT IS FOR GUIDANCE ONLY. IF USED AS PART OF A REVIEW PROCEDURE FOR PUBLICATION, IT SHOULD NOT REPLACE THE EXPERTISE OF AN EXPERIENCED CRYSTALLOGRAPHIC REFEREE.

No syntax errors found. CIF dictionary Interpreting this report

Datablock: jt-me3-1

Bond precision: C-C = 0.0015 A Wavelength=1.54184

Cell:	a=6.1149(2)	b=8.4497(3)	c=11.0343(4)
	alpha=90.472(3)	beta=101.250(3)	gamma=102.608(3)
Temperature:	103 K		

	Calculated	Reported
Volume	544.92(3)	544.92(3)
Space group	P -1	P -1
Hall group	-P 1	-P 1
Moiety formula	C11 H12 O6	C11 H12 O6
Sum formula	C11 H12 O6	C11 H12 O6
Mr	240.21	240.21
Dx,g cm-3	1.464	1.464
Z	2	2
Mu (mm-1)	1.035	1.035
F000	252.0	252.0
F000'	252.96	
h,k,lmax	7,10,13	7,10,13
Nref	2273	2223
Tmin,Tmax	0.827,0.876	0.780,1.000
Tmin'	0.800	

Correction method= # Reported T Limits: Tmin=0.780 Tmax=1.000
AbsCorr = GAUSSIAN

Data completeness= 0.978 Theta(max)= 75.442

R(reflections)= 0.0310(2190) wR2(reflections)= 0.0813(2223)

S = 1.053 Npar= 158

Compound **8**: CCDC Deposition Nubmer 2520617

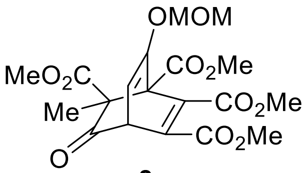

8

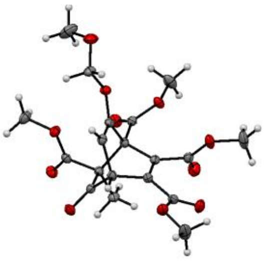

checkCIF/PLATON report

Structure factors have been supplied for datablock(s) jt-2-200-1

THIS REPORT IS FOR GUIDANCE ONLY. IF USED AS PART OF A REVIEW PROCEDURE FOR PUBLICATION, IT SHOULD NOT REPLACE THE EXPERTISE OF AN EXPERIENCED CRYSTALLOGRAPHIC REFEREE.

No syntax errors found. CIF dictionary Interpreting this report

Datablock: jt-2-200-1

Bond precision:	C-C = 0.0020 A		Wavelength=1.54184
Cell:	a=37.20872(15)	b=29.73371(15)	c=7.45609(3)
	alpha=90	beta=90	gamma=90
Temperature:	103 K		

	Calculated	Reported
Volume	8249.07(6)	8249.07(6)
Space group	F d d 2	F d d 2
Hall group	F 2 -2d	F 2 -2d
Moiety formula	C19 H22 O11	C19 H22 O11
Sum formula	C19 H22 O11	C19 H22 O11
Mr	426.37	426.36
Dx,g cm-3	1.373	1.373
Z	16	16
Mu (mm-1)	0.983	0.983
F000	3584.0	3584.0
F000'	3597.80	
h,k,lmax	46,37,9	46,37,9
Nref	4306[2324]	4291
Tmin,Tmax	0.825,0.888	0.930,1.000
Tmin'	0.819	

Correction method= # Reported T Limits: Tmin=0.930 Tmax=1.000
AbsCorr = MULTI-SCAN

Data completeness= 1.85/1.00 Theta(max)= 75.993

R(reflections)= 0.0223(4275) wR2(reflections)= 0.0585(4291)

S = 1.063 Npar= 277

Compound **21**: CCDC Deposition Number 2520616

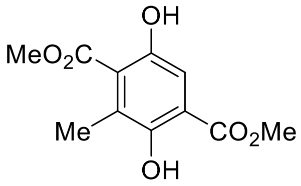

21

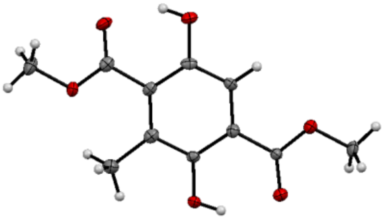

Datablock: jt-2-1xx-7

Bond precision: C-C = 0.0040 A Wavelength=1.54184

Cell:	a=18.6238(2)	b=8.1878(1)	c=6.9054(1)
	alpha=90	beta=90.109(1)	gamma=90
Temperature:	103 K		

	Calculated	Reported
Volume	1052.99(2)	1052.99(2)
Space group	C c	C 1 c 1
Hall group	C -2yc	C -2yc
Moiety formula	C11 H12 O6	C11 H12 O6
Sum formula	C11 H12 O6	C11 H12 O6
Mr	240.21	240.21
Dx,g cm-3	1.515	1.515
Z	4	4
Mu (mm-1)	1.072	1.072
F000	504.0	504.0
F000'	505.93	
h,k,lmax	23,10,8	23,10,8
Nref	2201[1103]	2130
Tmin,Tmax	0.920,0.965	0.914,1.000
Tmin'	0.880	

Correction method= # Reported T Limits: Tmin=0.914 Tmax=1.000
AbsCorr = MULTI-SCAN

Data completeness= 1.93/0.97 Theta(max)= 75.597

R(reflections)= 0.0323(2083) wR2(reflections)= 0.0943(2130)

S = 1.086 Npar= 159

8. References.

1) T. S. Alexander, T. J. Clay, B. Maldonado, J. M. Nguyen and D. B. C. Martin, *Tetrahedron* **2019**, *75*, 2229–2238.

9. NMR spectra.

^{1}H NMR of **5**

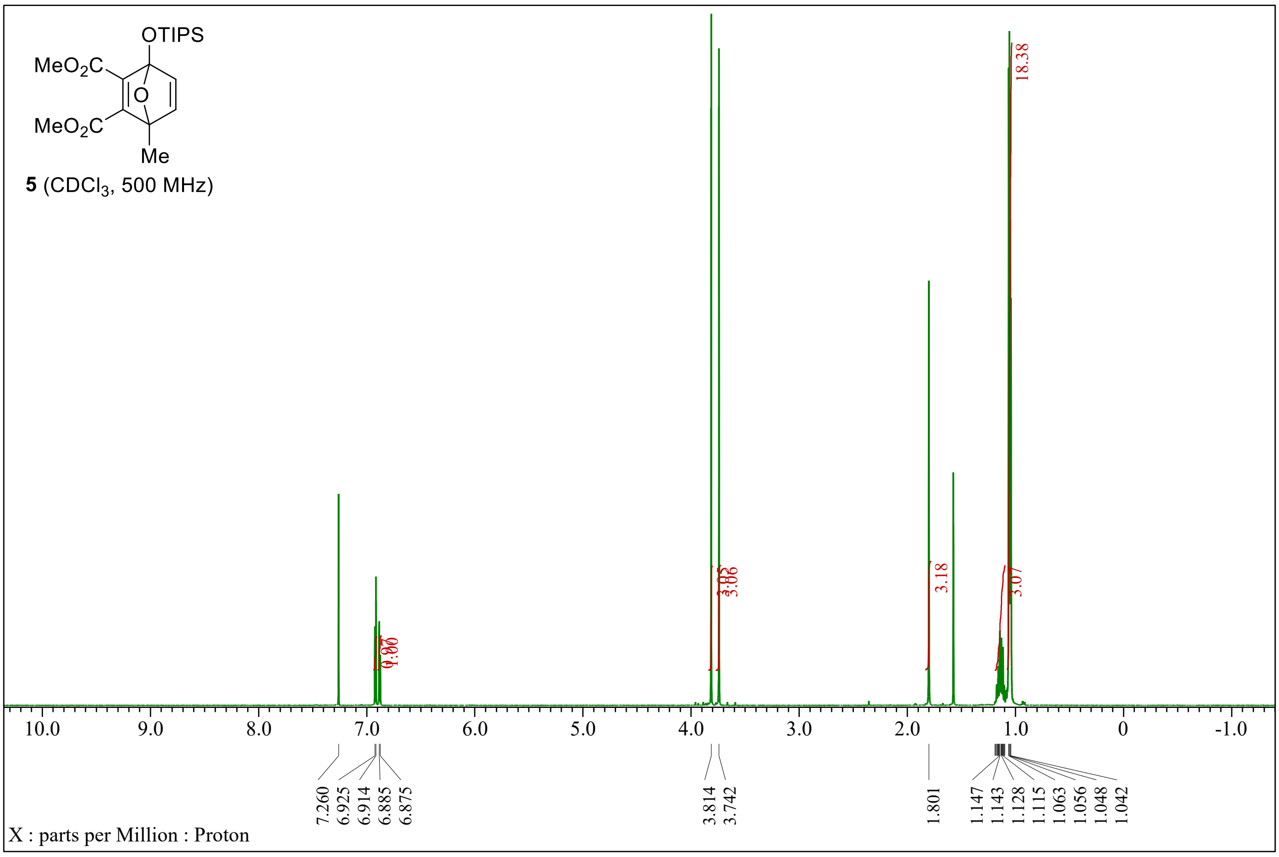

^{13}C NMR of **5**

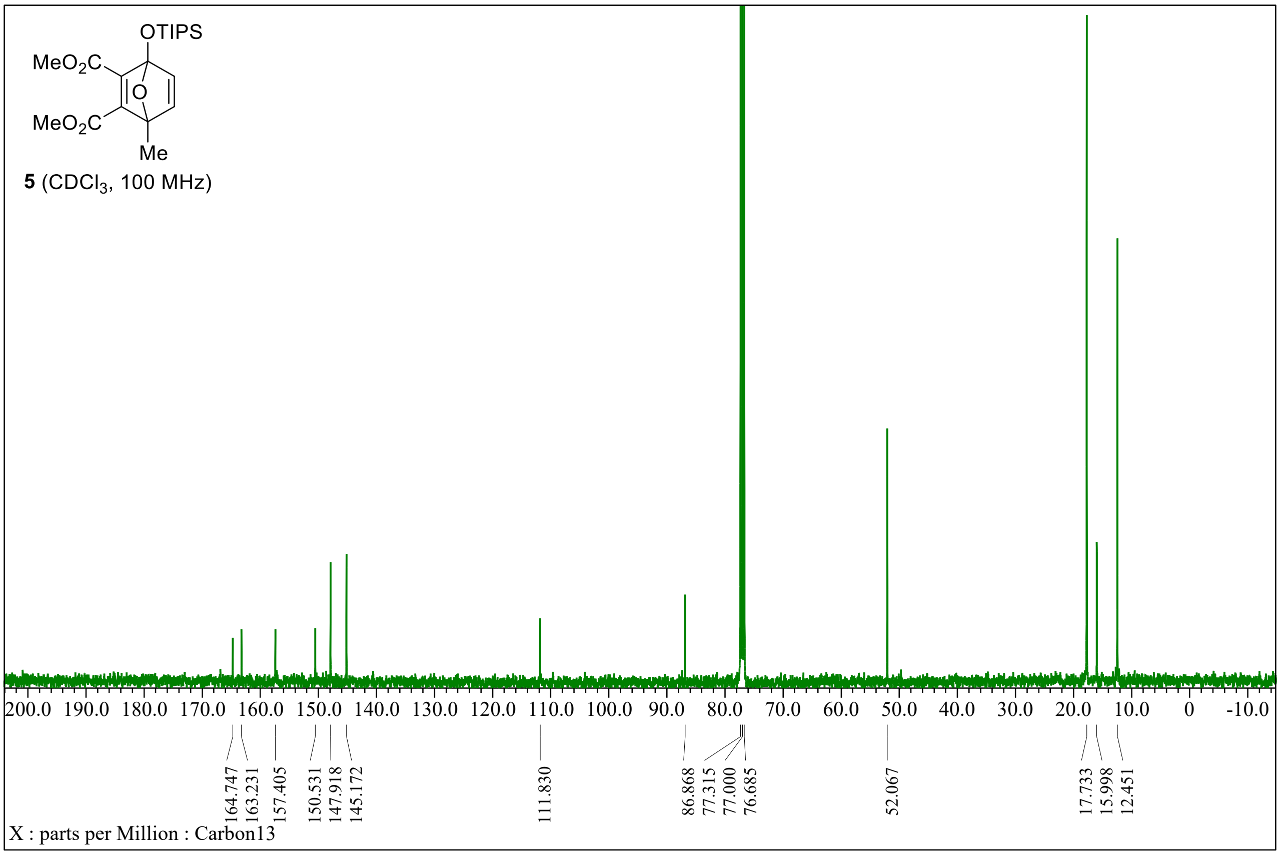

^{1}H NMR of **6**

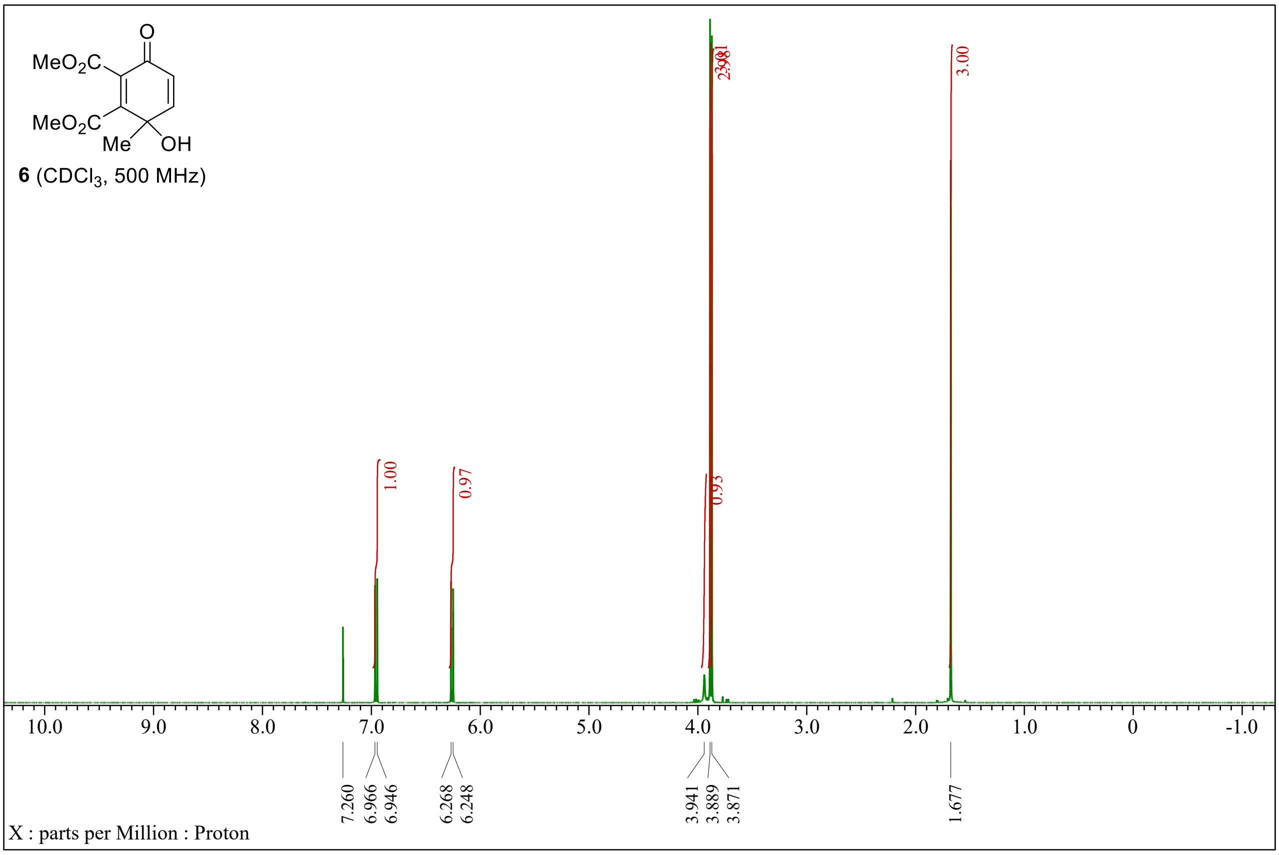

^{13}C NMR of **6**

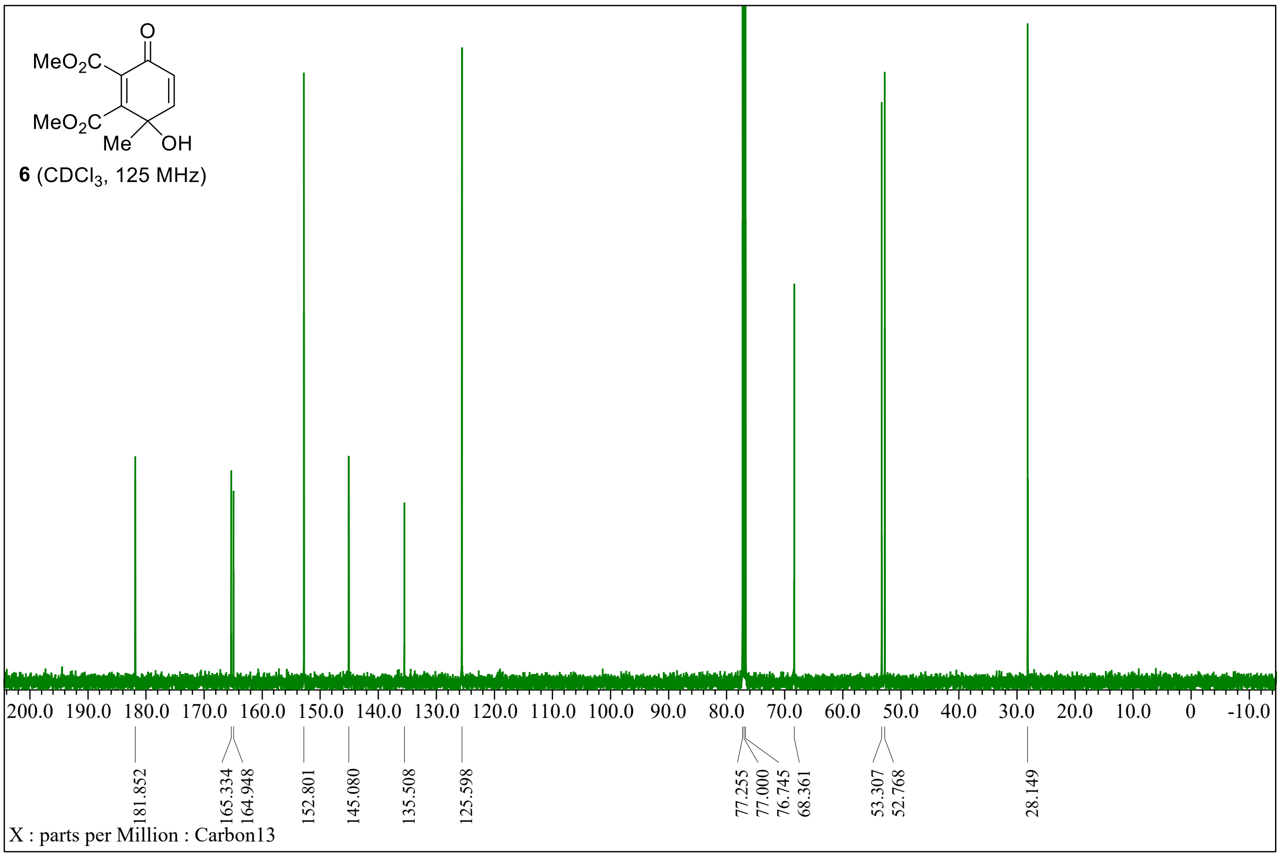

^{1}H NMR of **7**

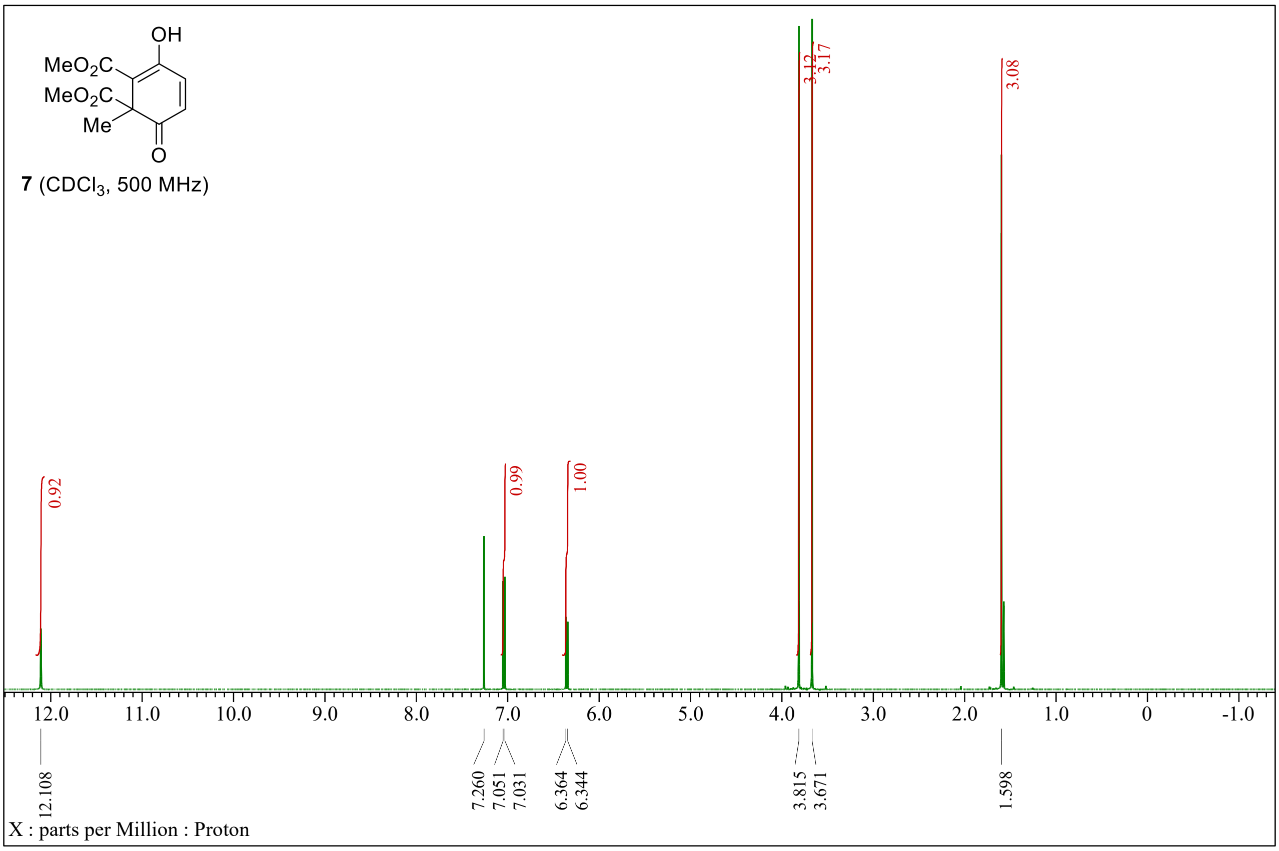

^{13}C NMR of **7**

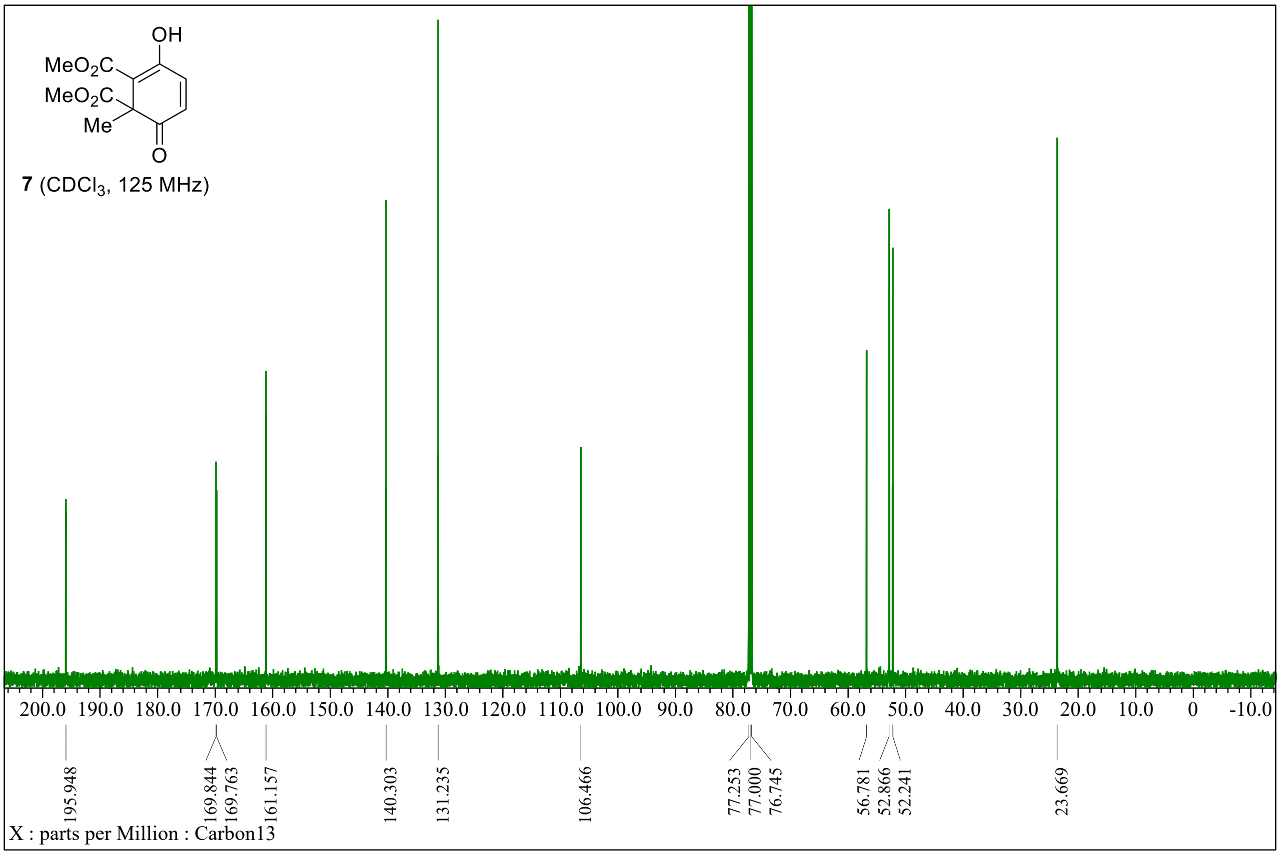

^{1}H NMR of **10**

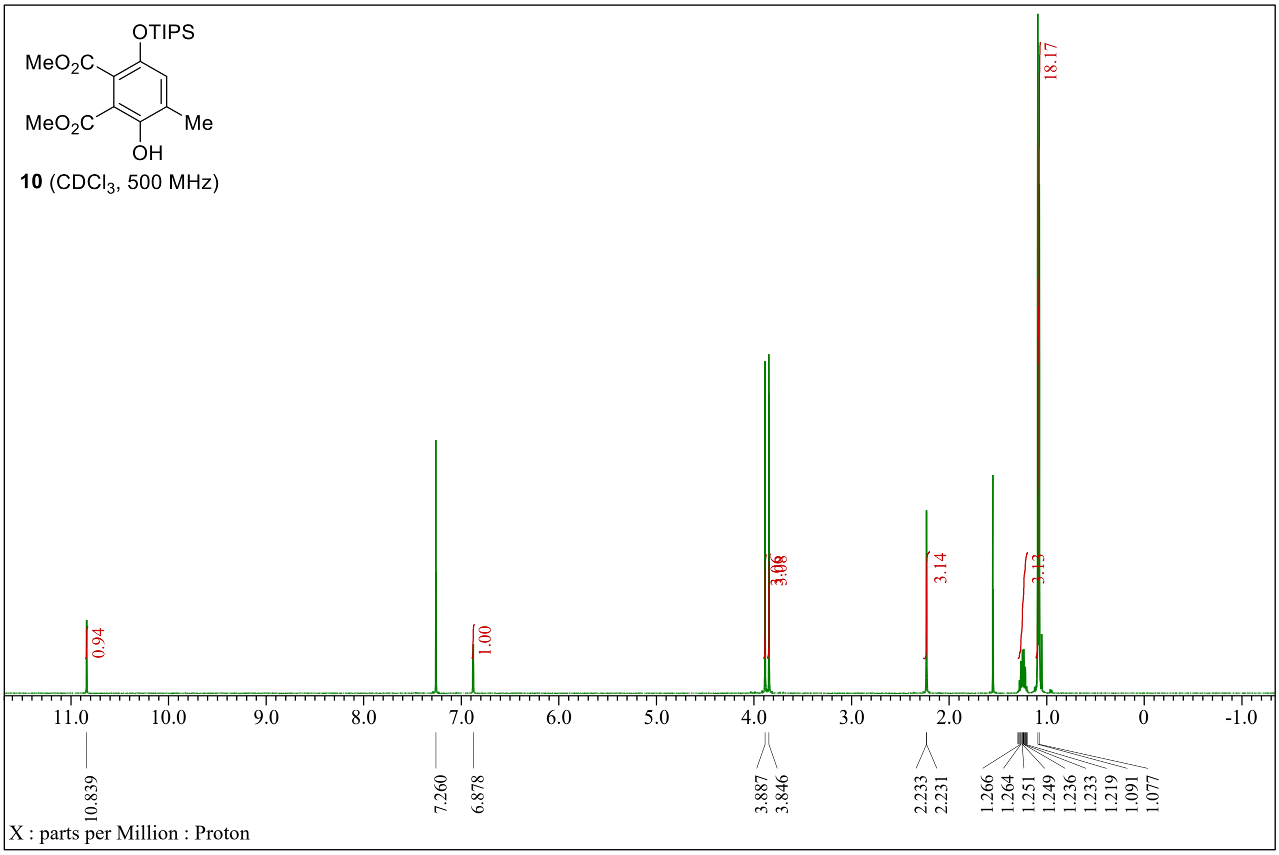

^{13}C NMR of **10**

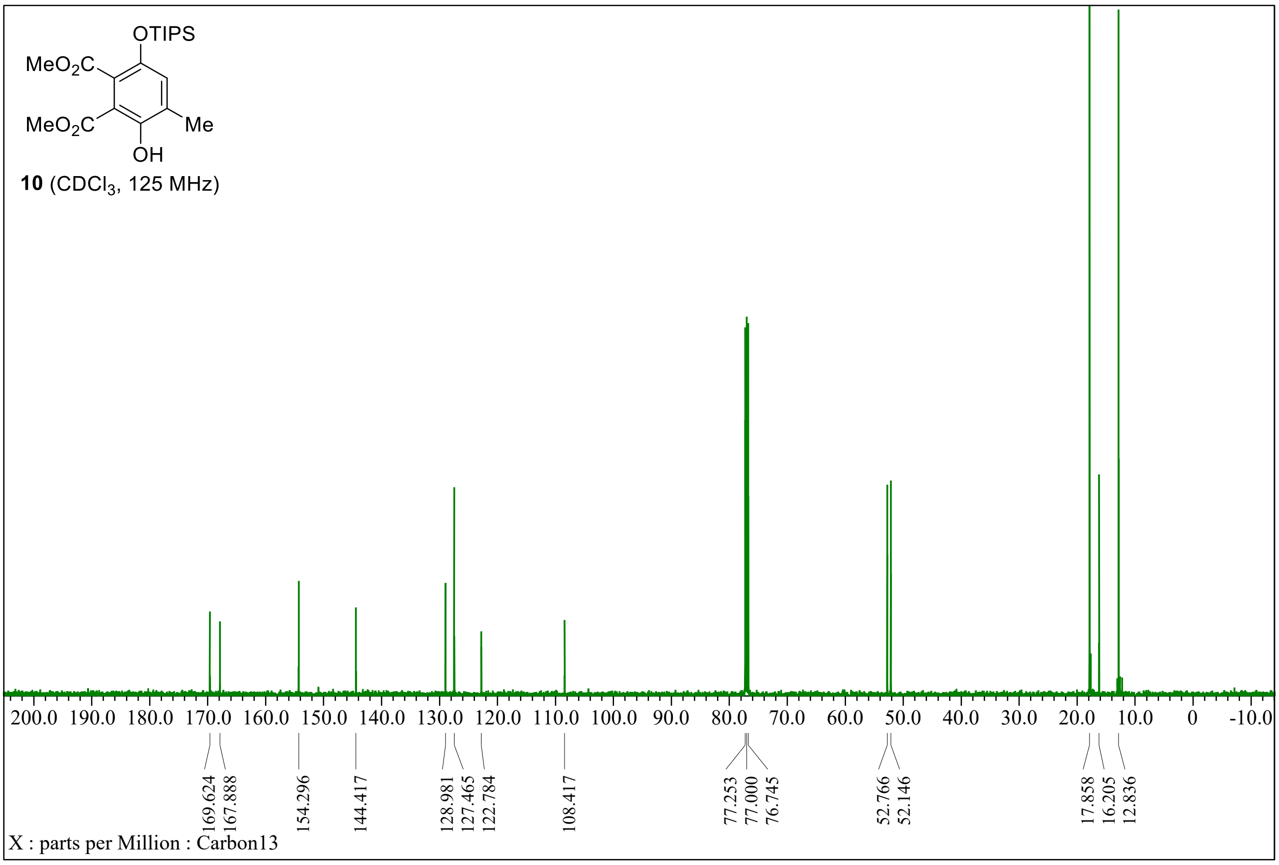

^{1}H NMR of **16**

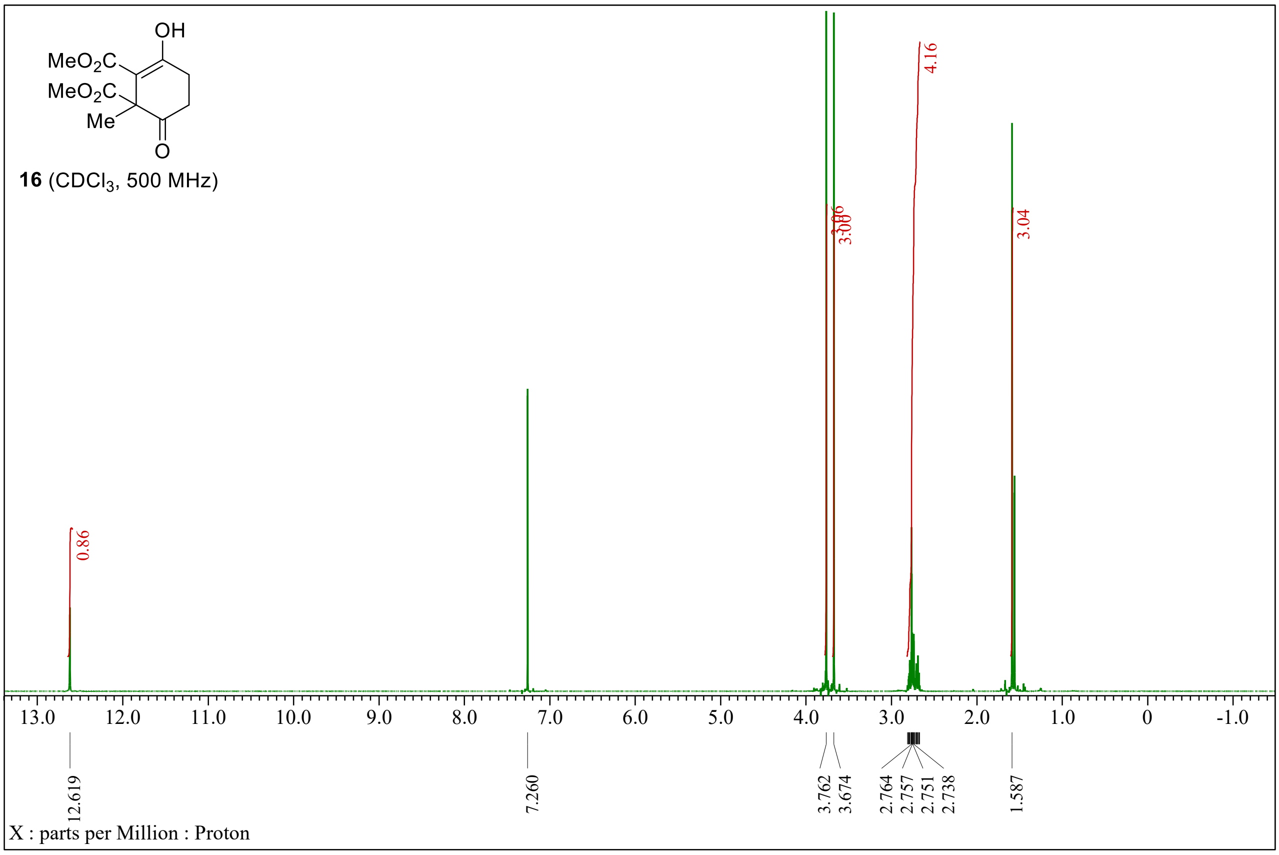

^{13}C NMR of **16**

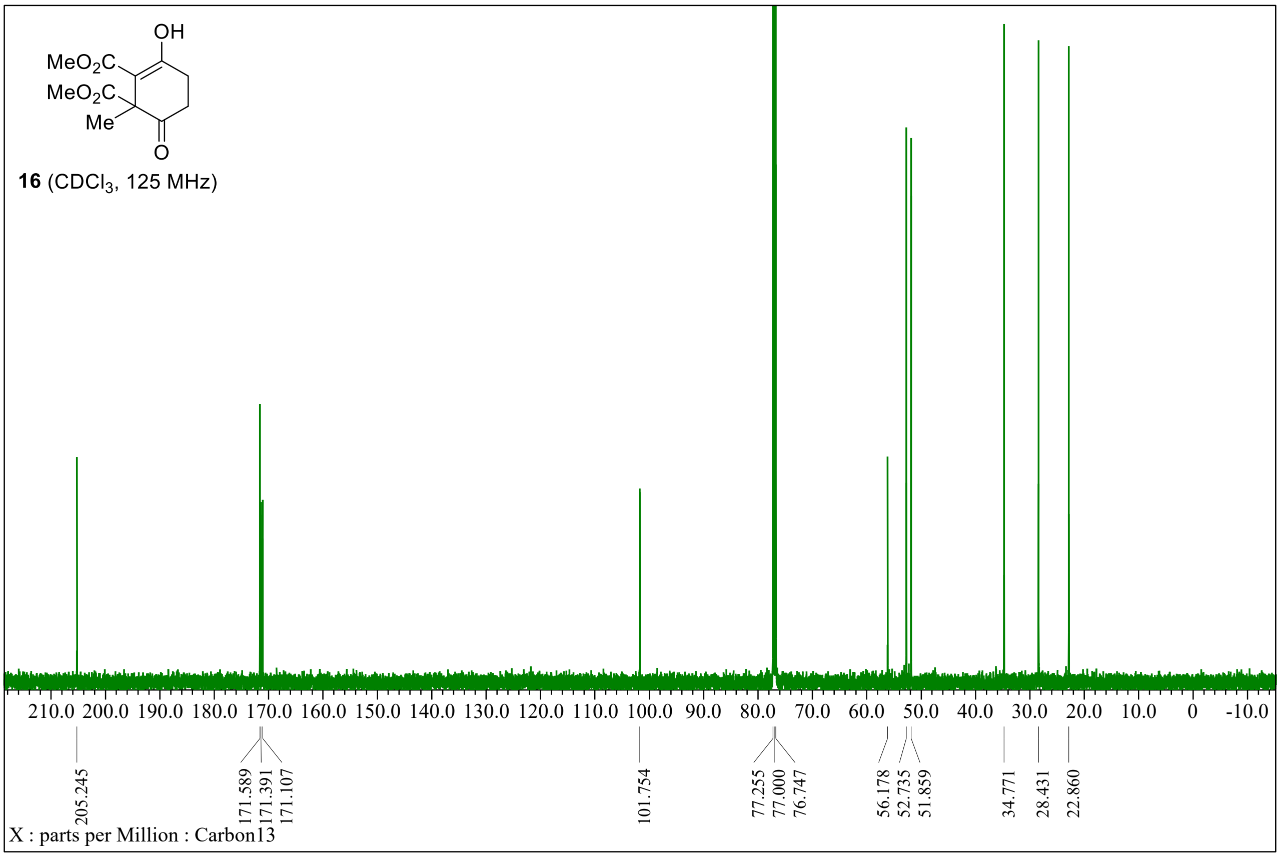

^{1}H NMR of **17**

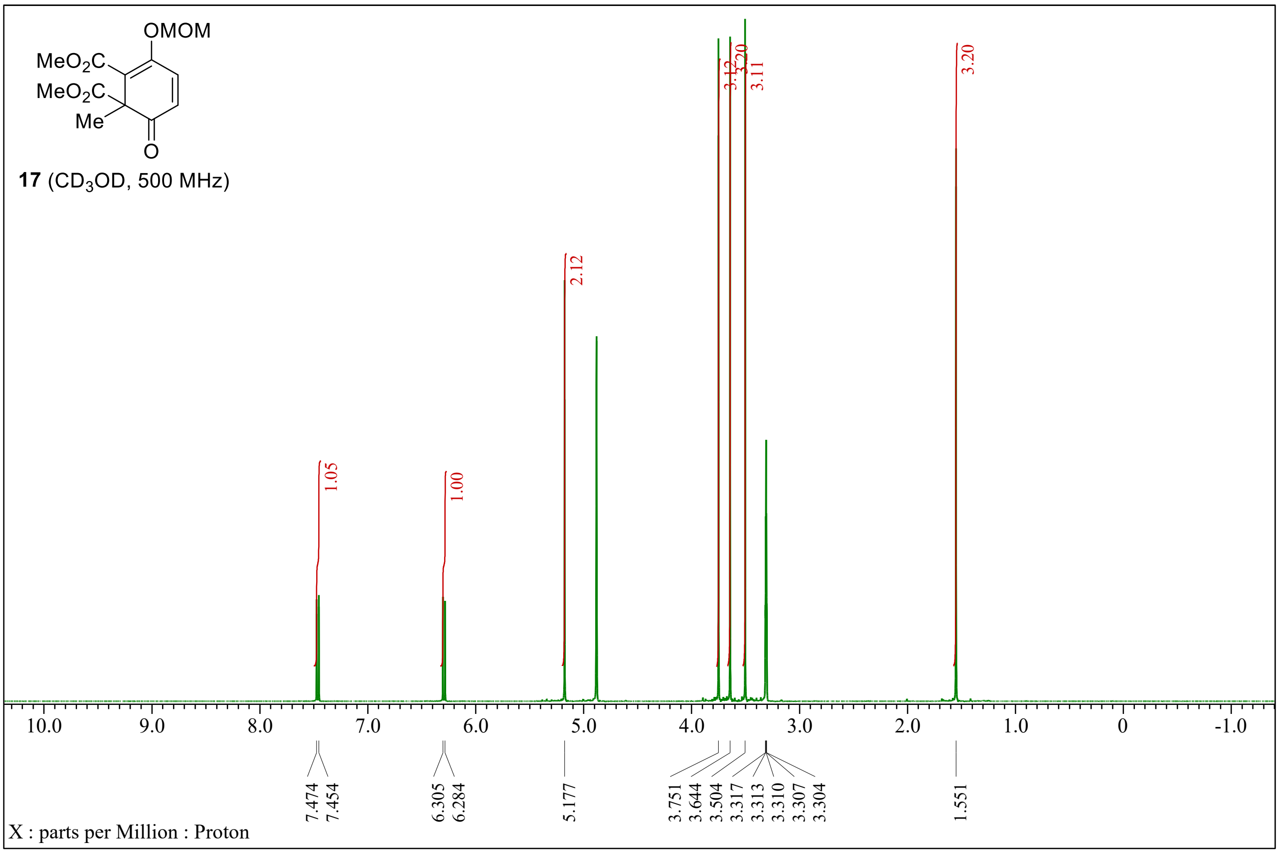

^{13}C NMR of **17**

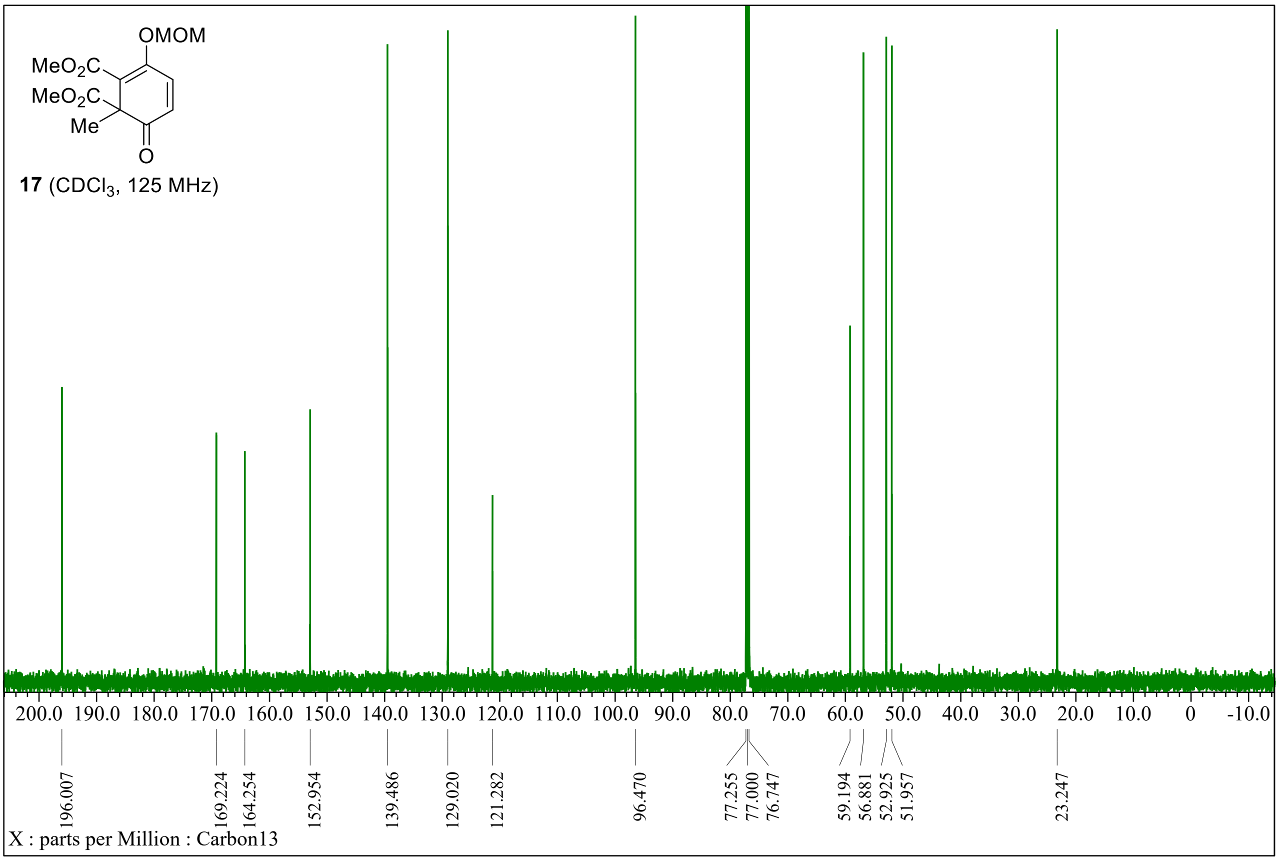

^{1}H NMR of **8**

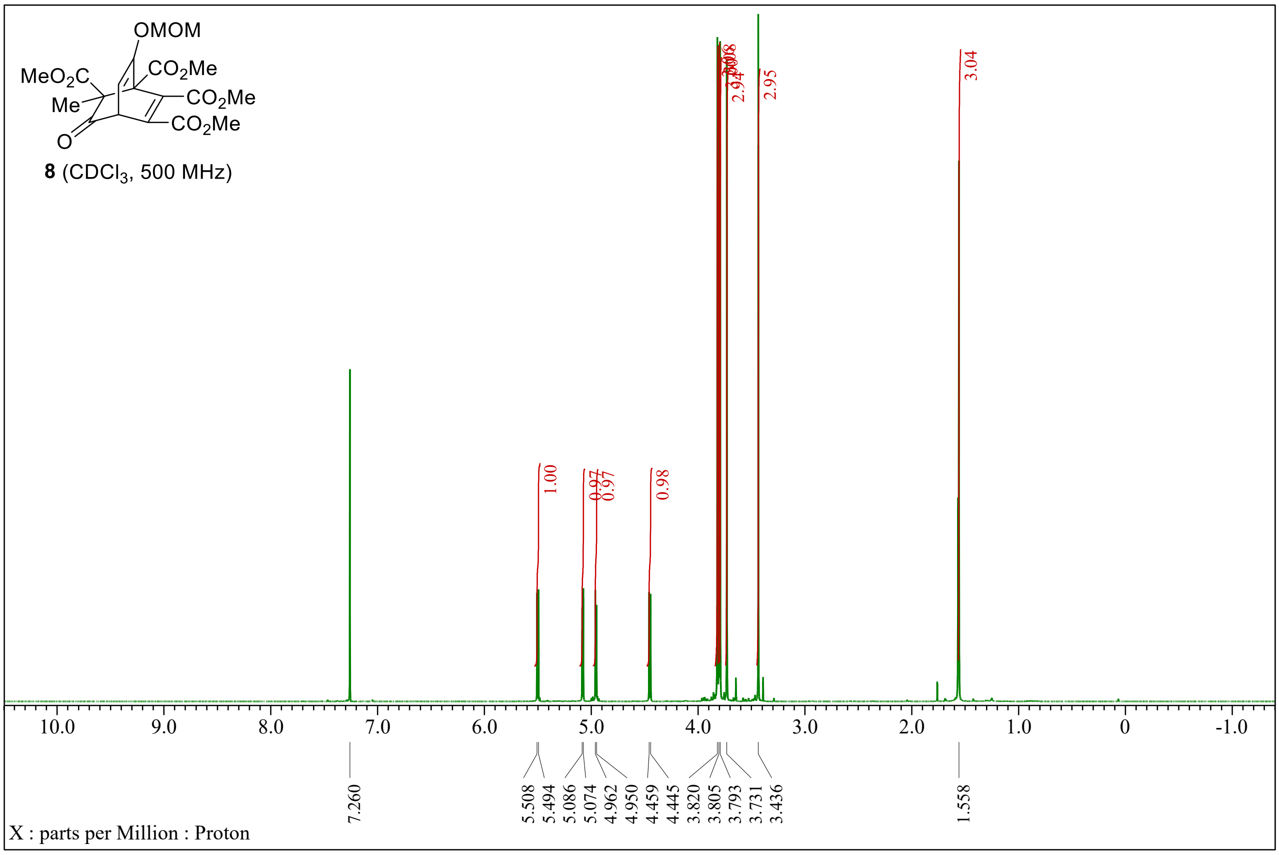

^{13}C NMR of **8**

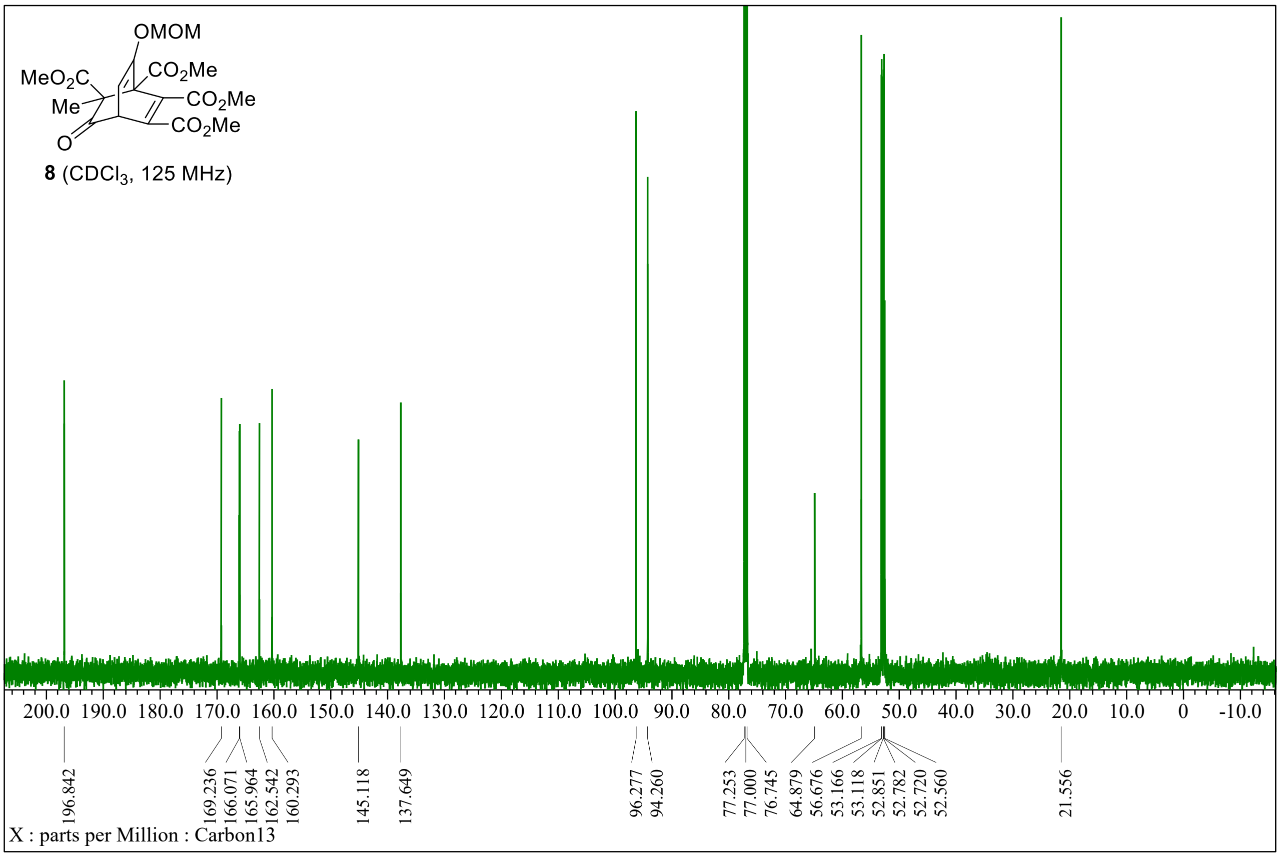

^{1}H NMR of **18**

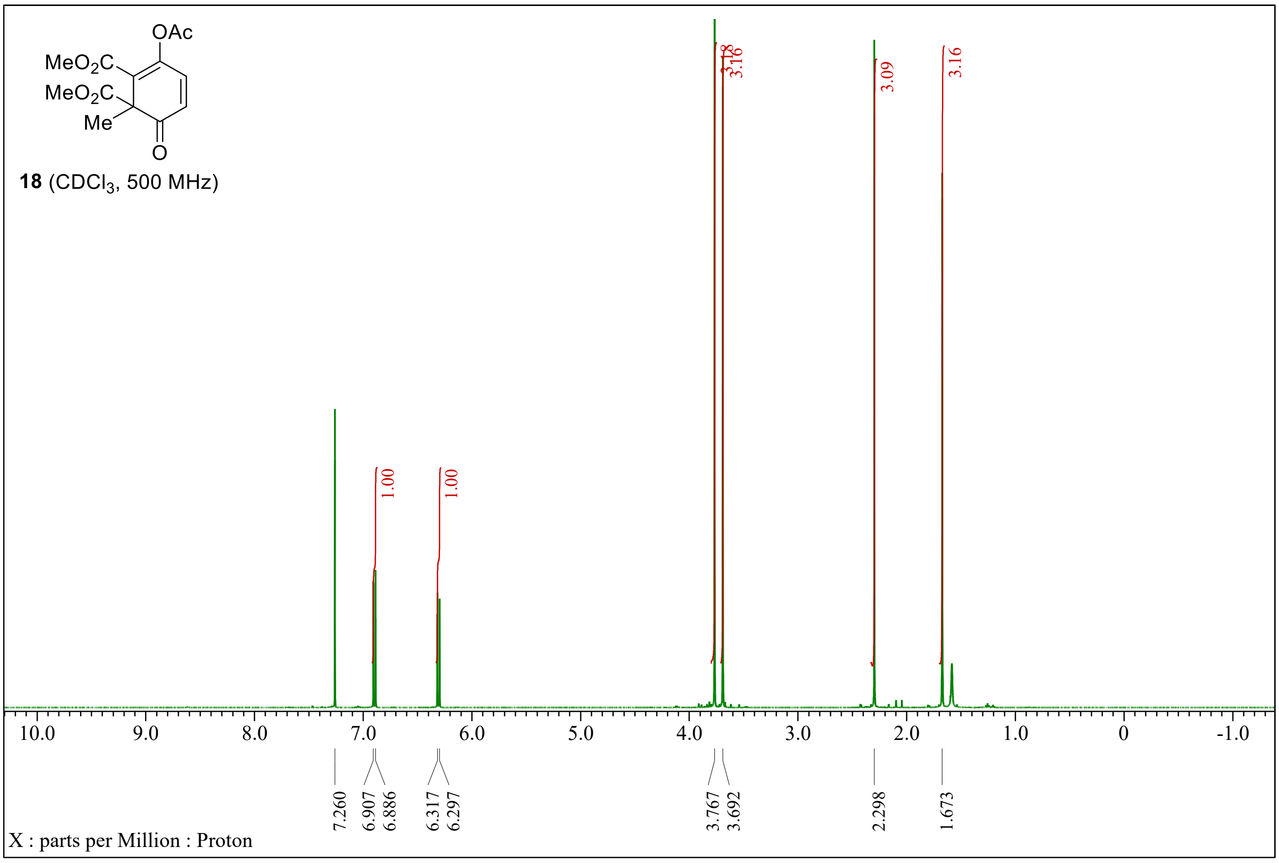

^{13}C NMR of **18**

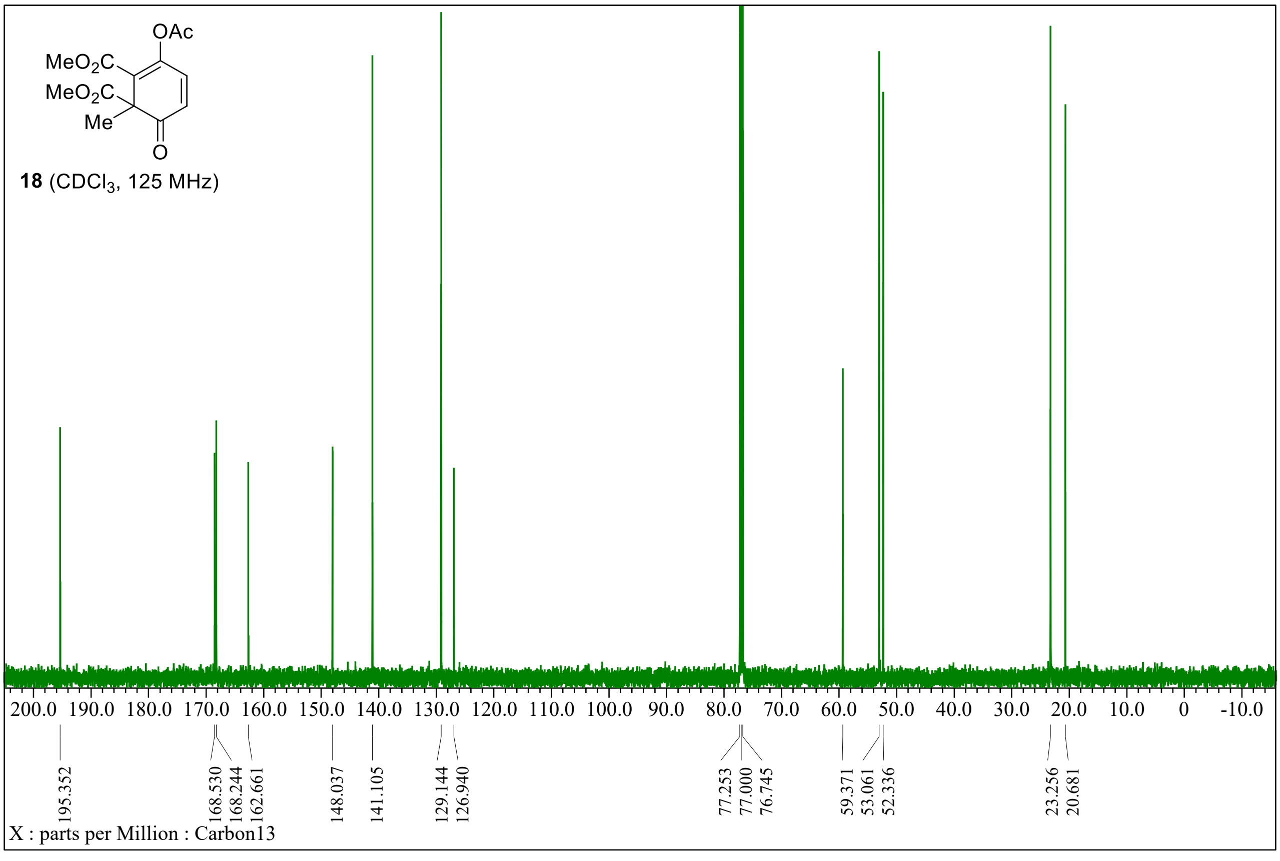

^{1}H NMR of **19-major**

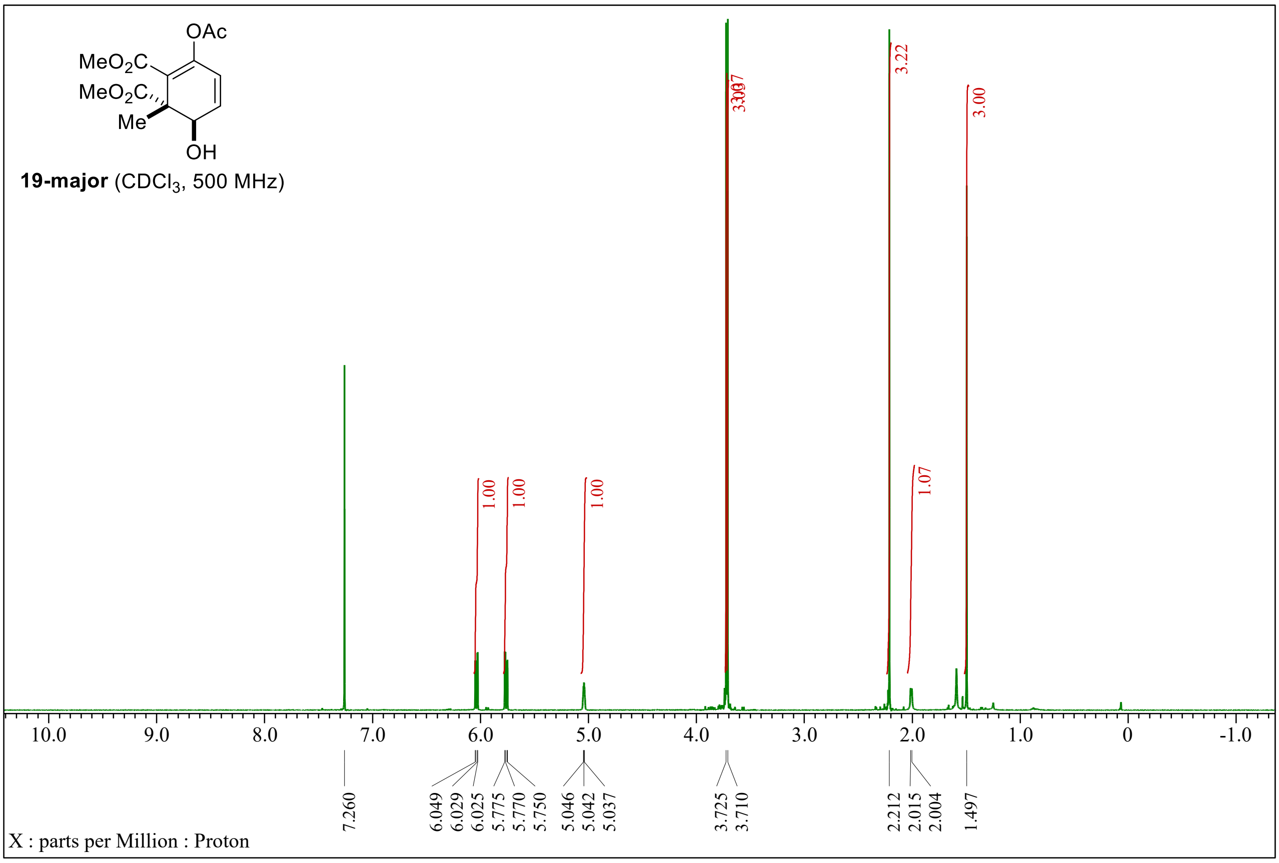

^{13}C NMR of **19-major**

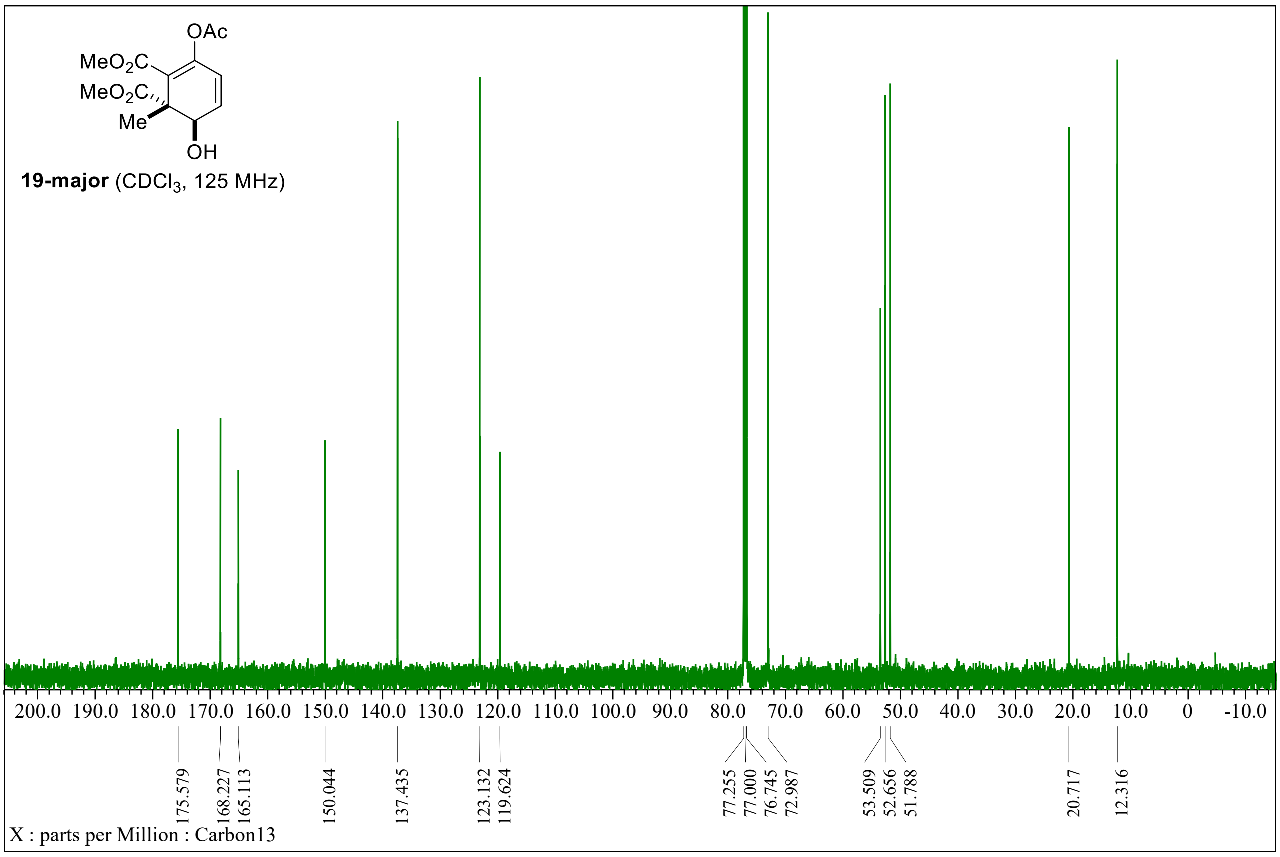

^{1}H NMR of **19-minor**

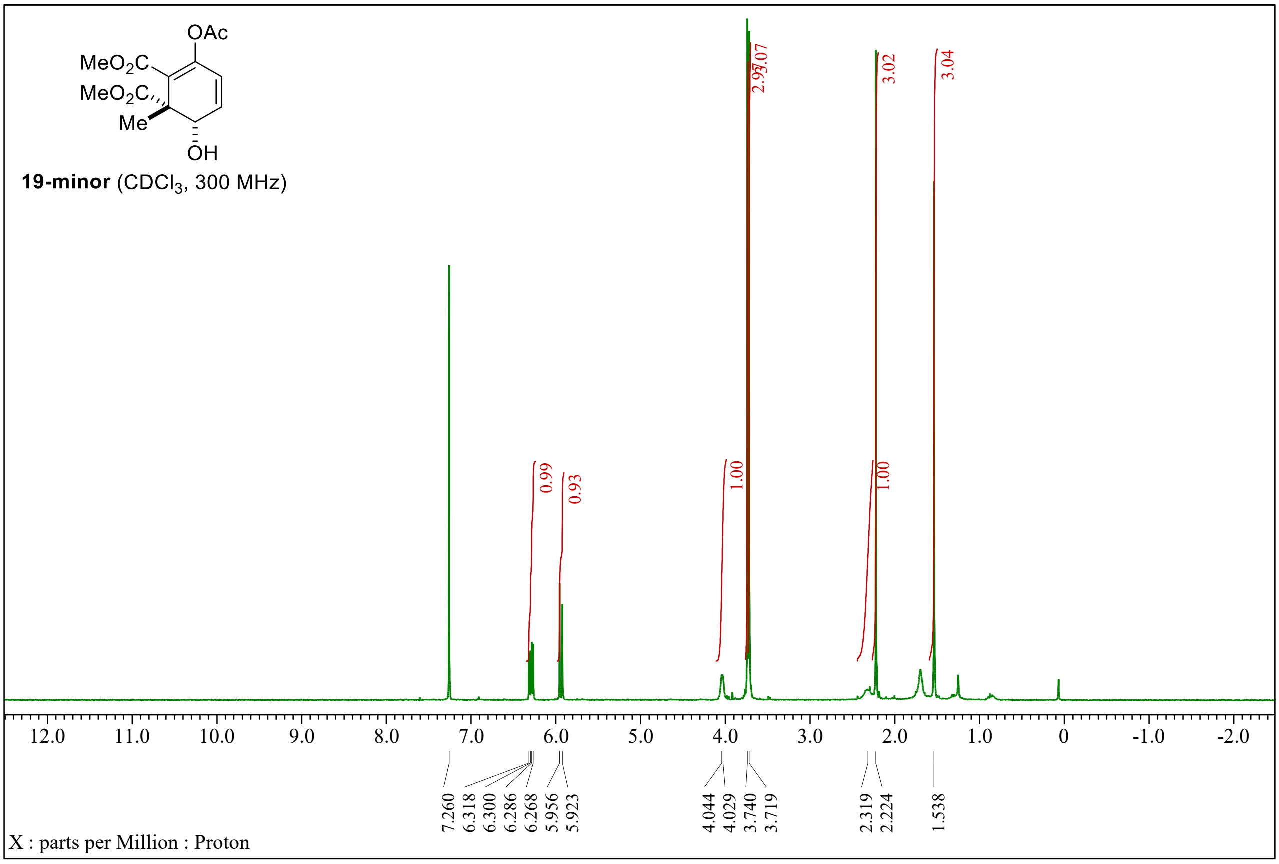

^{13}C NMR of **19-minor**

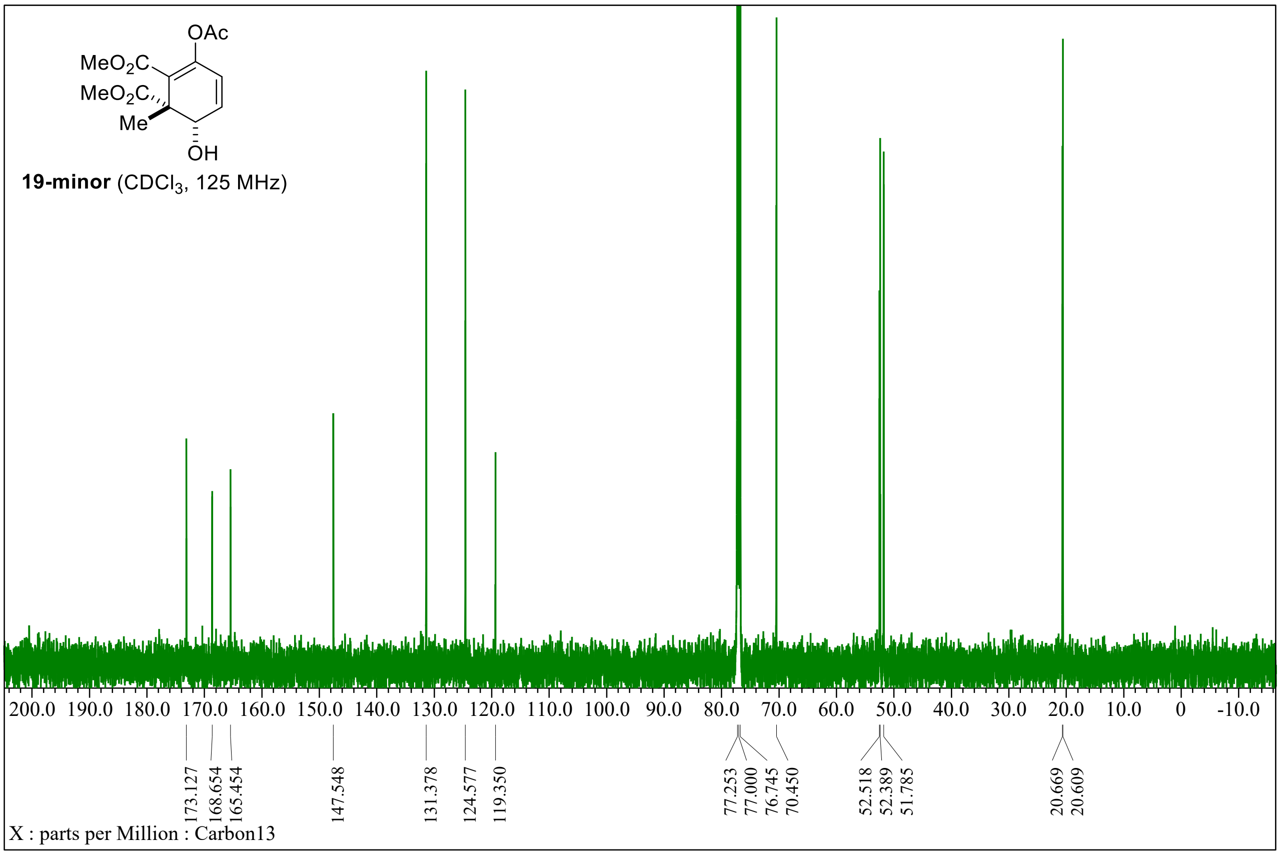

NOESY of **19-minor**

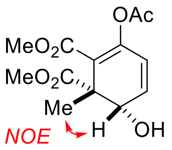

19-minor ($CDCl_3$, 300 MHz)

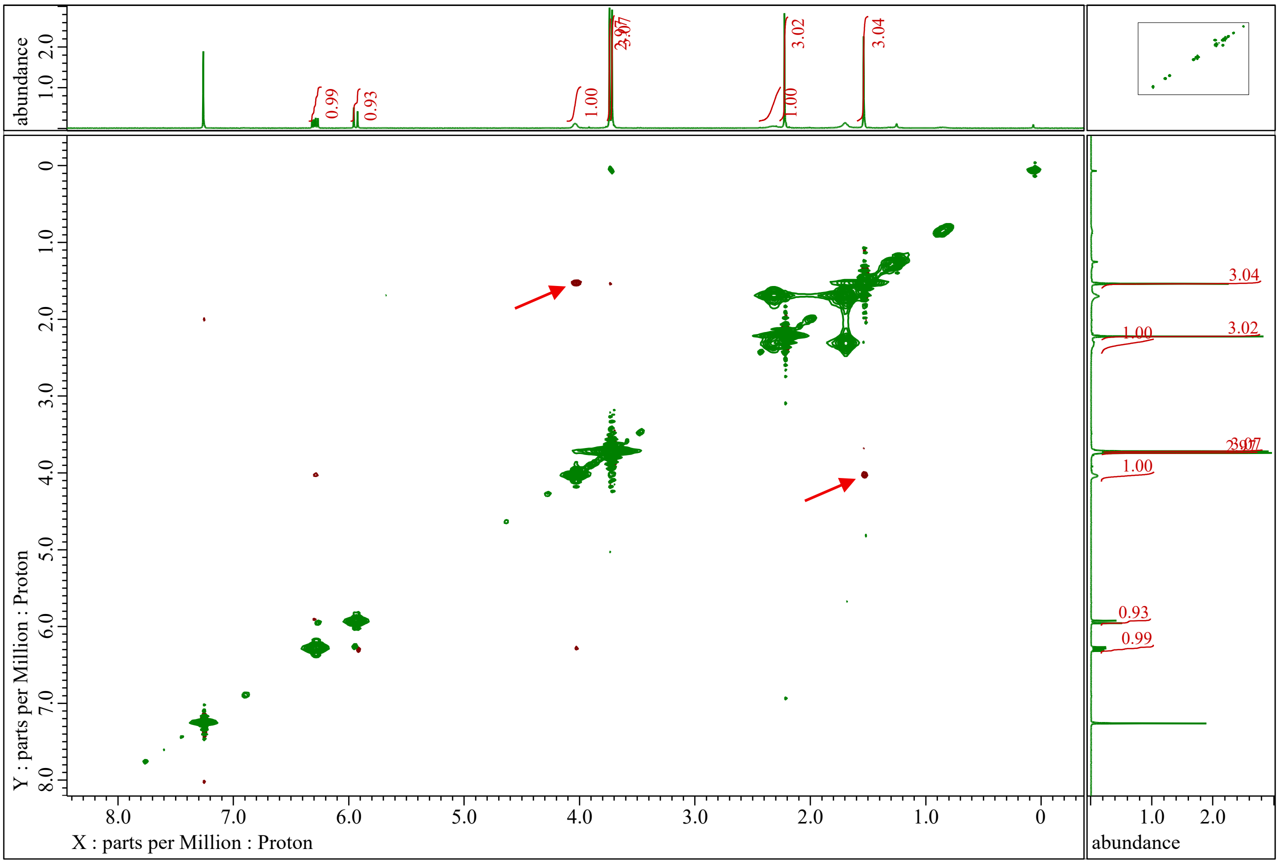

^{1}H NMR of **20**

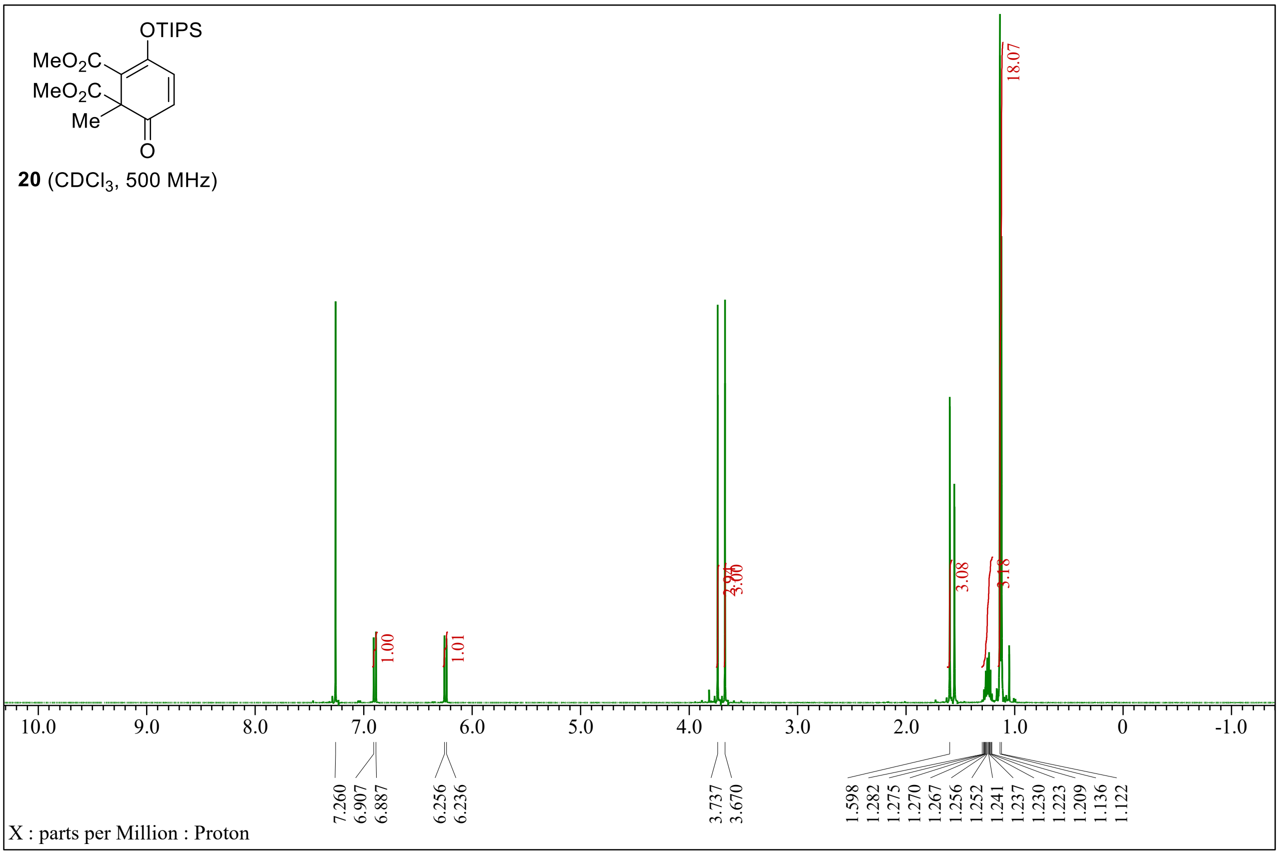

^{13}C NMR of **20**

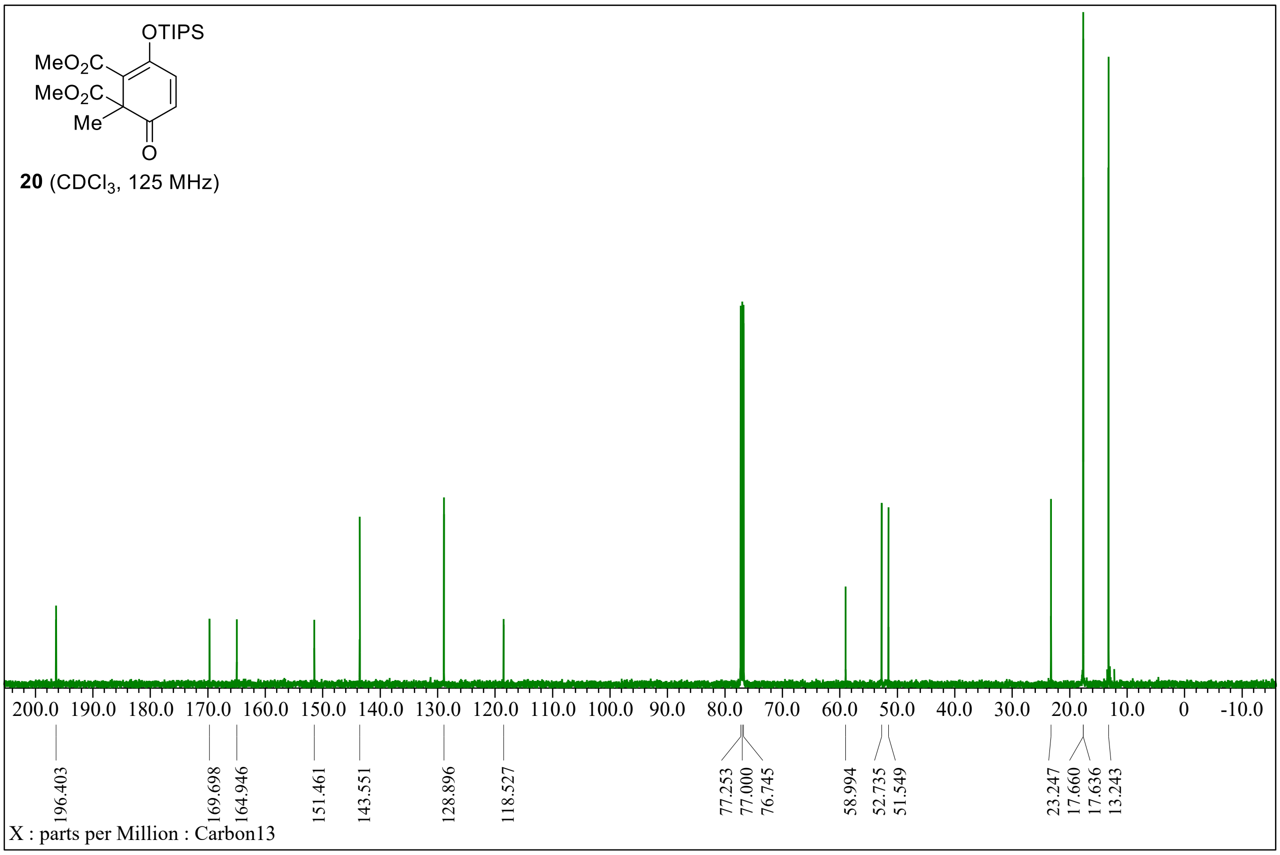

^{1}H NMR of **9**

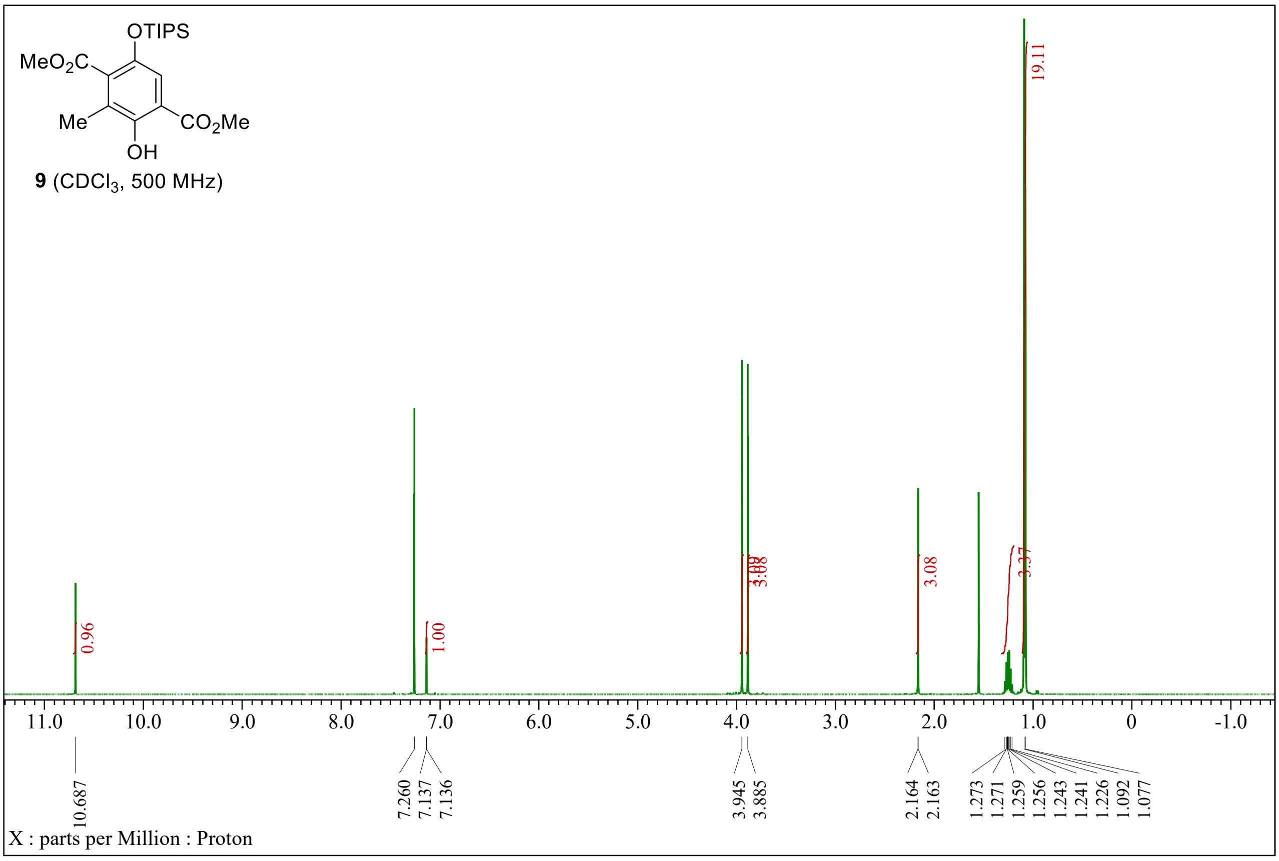

^{13}C NMR of **9**

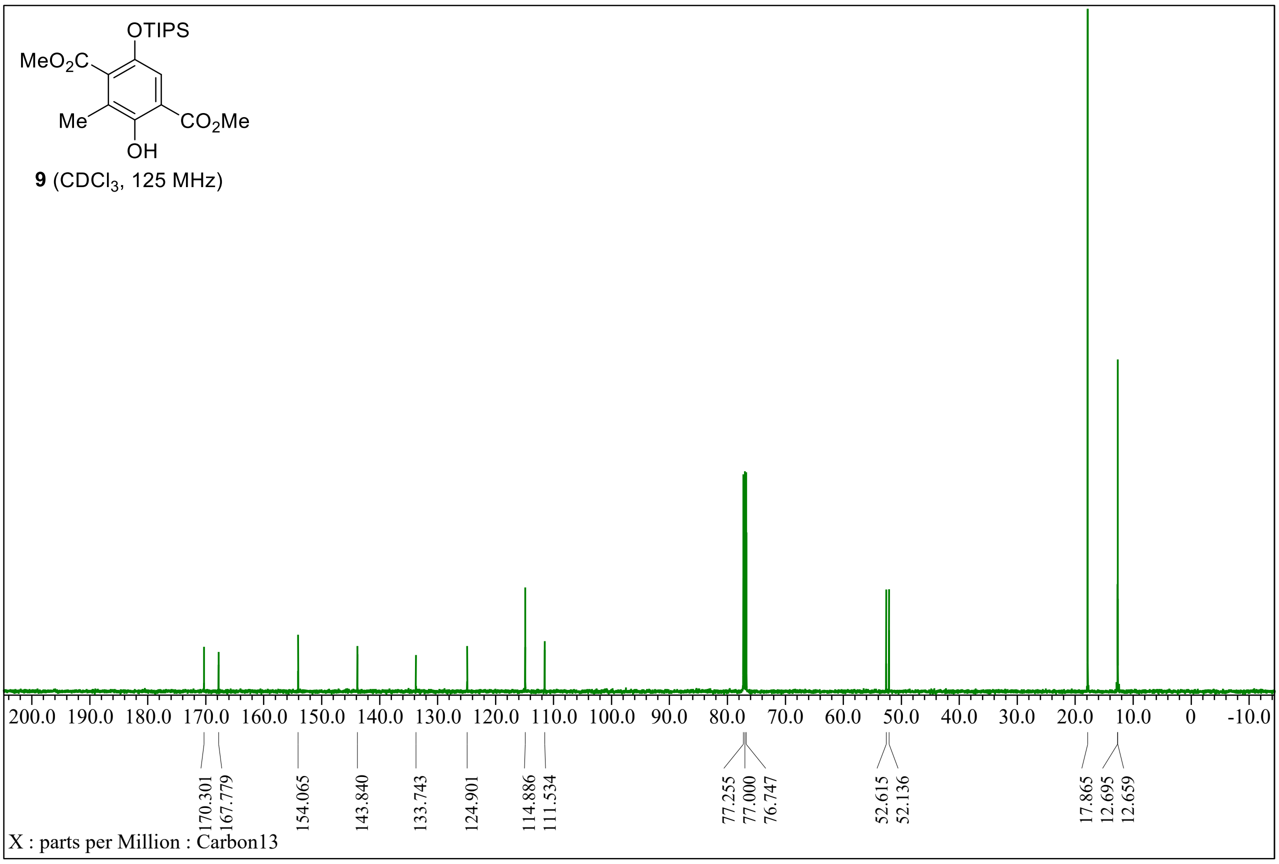

^{1}H NMR of **21**

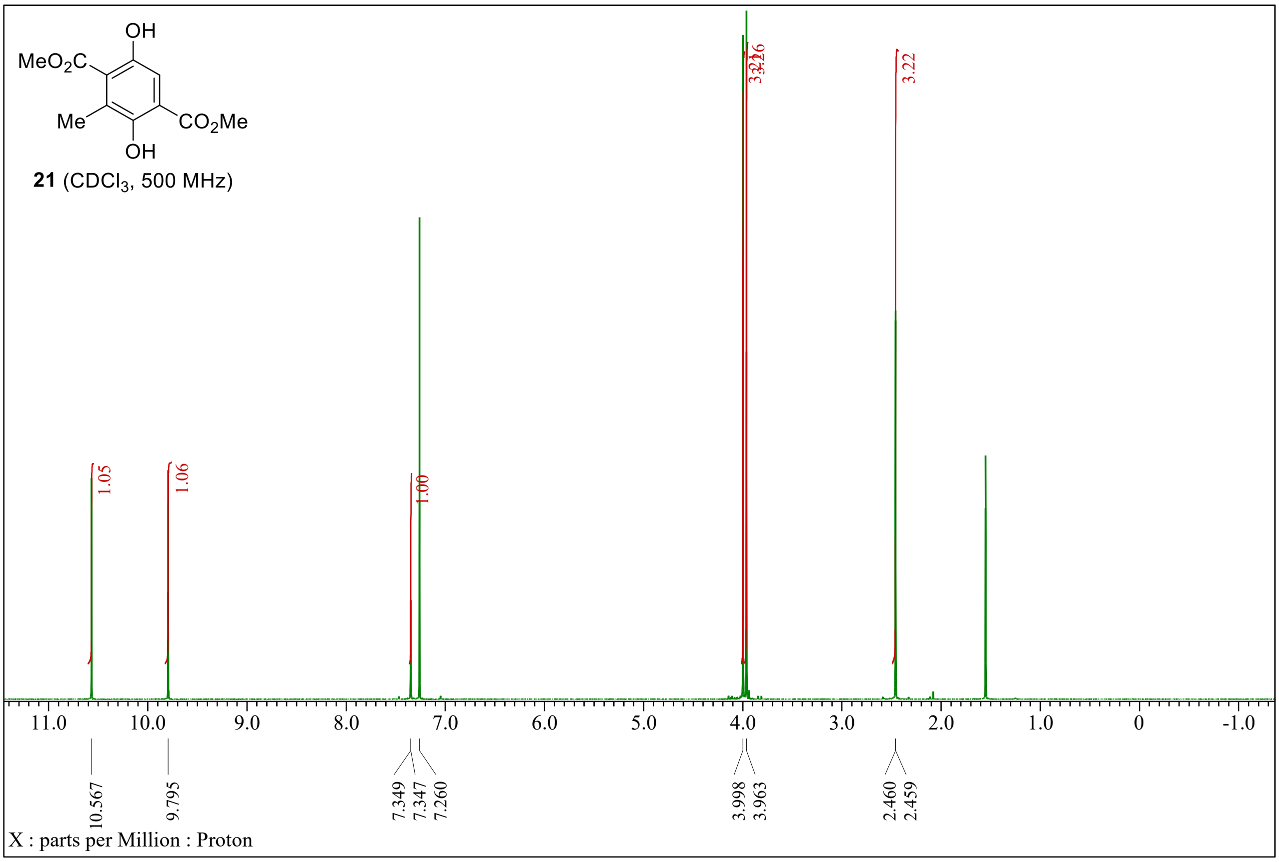

^{13}C NMR of **21**

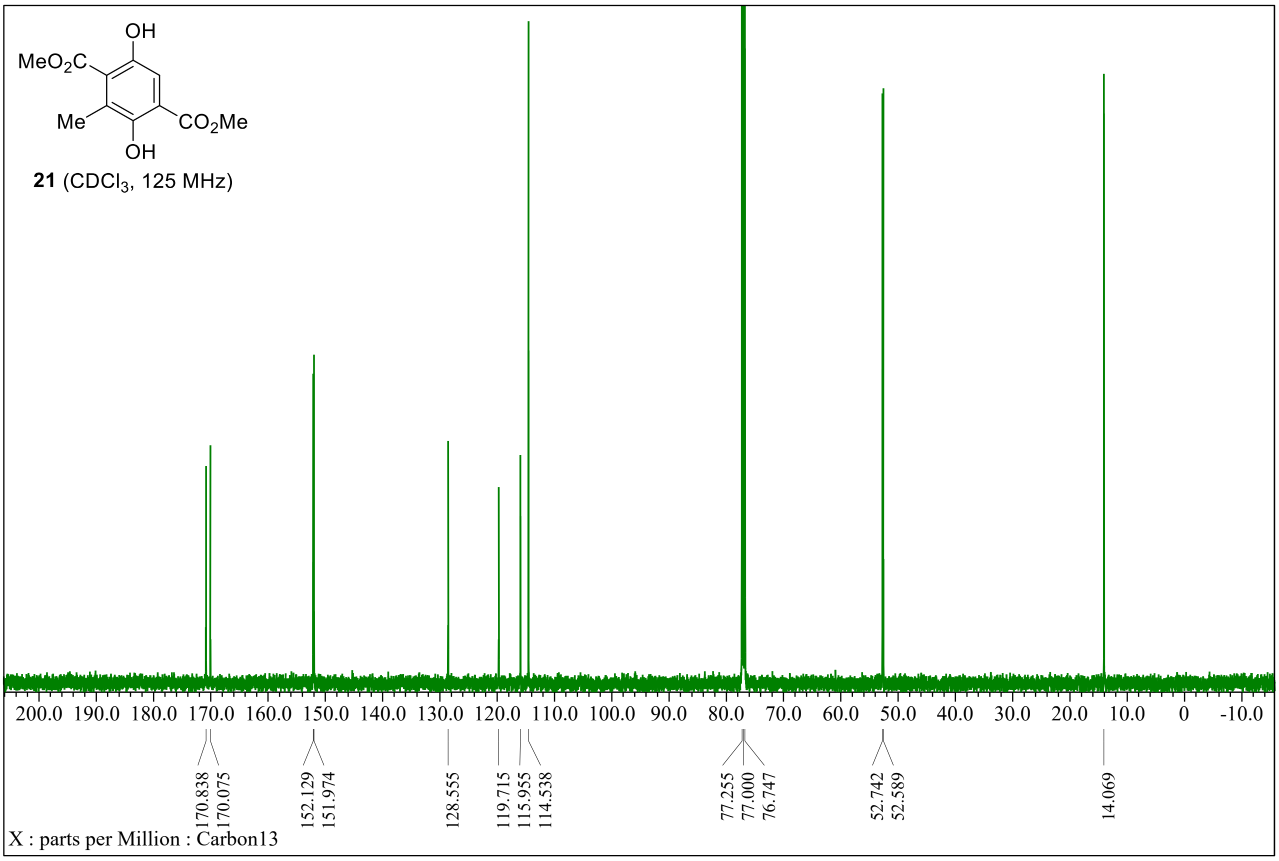

Supplement: RA-016-D6RA01853J-s001 [file RA-016-D6RA01853J-s001.pdf]
